# Mechano-ID: Proximity Labeling of Mechanically Active Receptors Reveals the Mechanome and Tags Mechanically Active Cells

**DOI:** 10.1021/jacs.5c05203

**Published:** 2025-09-26

**Authors:** Rong Ma, Mohamed Husaini Bin Abdul Rahman, Christian M. Beusch, Brendan R. Deal, David E. Gordon, Khalid Salaita

**Affiliations:** 1 Department of Chemistry, 1371 Emory University, 1515 Dickey Drive, Atlanta, Georgia 30322, United States; 2 Wallace H. Coulter Department of Biomedical Engineering, Emory University and Georgia Institute of Technology, 313 Ferst Drive NW, Atlanta, Georgia 30332, United States; 3 Pathology Advanced Translational Research Unit (PATRU), Department of Pathology and Laboratory Medicine, Emory University School of Medicine, 1462 Clifton Road, Atlanta, Georgia 30322, United States; 4 Department of Surgical Sciences, Uppsala University, Dag Hammarskjölds väg 7, Uppsala 752 37, Sweden

## Abstract

A major challenge in the field of mechanobiology relates to the lack of methods that enable the identification of mechanically active receptors, associated proteins, and the individual cells that display enhanced force generation. For example, potent T cell activation requires the transmission of biophysical forces between the T cell receptor (TCR) and its peptide-loaded major histocompatibility (pMHC) complex antigens. Interestingly, TCR-antigen interactions are highly dynamic, displaying a broad range of force magnitudes between different cells and even within the same cell. Therefore, methods that can chemically tag mechanically active T cells, TCRs, and their associated proteomes, or mechanomes are highly desirable. Such techniques may enable a deeper understanding of the mechanisms governing immune responses and may also have broad applications in immunotherapy. Herein, we report a technique dubbed mechano-ID, which allows for mechanically selective proximity tagging by leveraging DNA-based molecular force probes that recruit proximity tagging enzymes. We demonstrate mechano-ID tagging of T cells using microscopy and flow cytometry, with further confirmation by proteomics and Western blotting of mechanically active T cell receptors.

## Graphical abstract

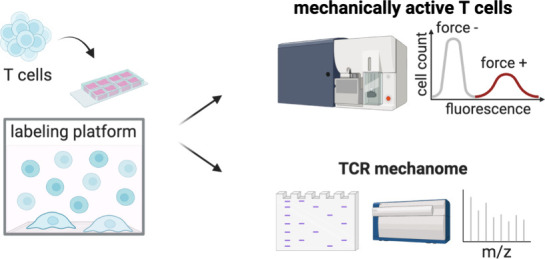

## Introduction

Over the past decade, the advent of molecular force probes has enabled the study of mechanotransduction, or the coupling between mechanical forces and biochemical signaling, at the cellular and molecular level.^1–8^ DNA force probes, in particular, have allowed for imaging of piconewton (pN) forces associated with different processes including cell adhesion and migration, as well as immune response and platelet activation.^5,9–12^ These probes generate a fluorescence signal in response to pN forces, enabling the mapping and quantification of cell forces. A major limitation of microscopy-based analysis of molecular forces pertains to the limited throughput of hundreds of measurements per experiment. For example, flow cytometry can perform a single cell analysis of protein and nucleic acid expression of millions of cells in a single experiment. Moreover, molecular tension probes combined with immunostaining cannot identify unknown proteins that may mediate mechanotransduction, i.e., the mechanome. These capabilities are particularly needed in mechanoimmunology, where hundreds of cell surface receptors interact with their ligands, transmitting forces at the junction between immune cells and their target cells to facilitate the recognition of cancer cells or infected cells. Therefore, new methods to study complex mechanical interactions and identify mechanically active cells and receptors without the requirement for microscopy are highly desired. Techniques to detect protein–protein interactions (PPI) at cell junctions^13–15^ are not sufficiently specific since many strong PPIs are not associated with force transmission and conversely weak affinity interactions may strengthen under mechanical load.^16^ This creates a grand challenge in developing tools to identify the ligand–receptor pairs mediating mechanical interactions and distinguishing the proteome associated with biophysical forces from static receptor–ligand binding.

One hallmark example of a receptor that displays mechanical sensitivity is the T cell receptor (TCR), which is central to antigen recognition and subsequent T cell activation.^17^ TCRs recognize antigenic peptides presented by the major histocompatibility complex (pMHC) on the membrane of target cells. With an estimated repertoire of up to ∼10^8^ diverse TCRs, T cells can recognize threatening peptides up to 10^9^ possible antigens.^18^ One growing model explaining TCR specificity and sensitivity is the mechanosensor model, which suggests that TCR-pMHC forces contribute to TCR activation.^19–21^ DNA force probes confirmed that T cells transmit pN forces to the TCR-pMHC complex.^10,22^ Interestingly, when altered peptide ligands (APL) with point mutations were presented to T cells in place of the cognate antigen, the TCR mechanical sampling of APLs correlated with the ligand potency, despite each APL having a similar affinity to the TCR.^11^

Taken together, the force-dependent nature of T cell activation leads us to an important question of whether we can identify the mechanically active T cells and TCR mechanomes, as they are indispensable during T cell activation and potent immune responses.^23^ The literature indicates that bond lifetimes range from hundreds of milliseconds to seconds,^24^ and hence, a rapid and biocompatible reaction with mechanical selectivity is required for mechanical tagging.^25^

One technique developed to tag transient PPI quickly and reliably is proximity tagging.^26^ This approach uses enzymes to generate reactive intermediates to covalently react with nearby proteins of interest, typically within tens of nanometers or less depending on the diffusion coefficient and half-life of the reactive intermediate.^27–29^ To date, peroxidases are one of the most used enzymes for proximity tagging because of their robustness and the wide availability of reagents. This class of enzymes catalyzes one electron oxidation of phenol derivatives to generate reactive phenoxyl radicals that attack exposed aromatic side chains (mainly tyrosines) on proximal proteins.^30,31^ Usually, the free radical substrate contains a biotin moiety for the easy isolation and downstream identification of labeled proteins. Peroxidases have demonstrated efficient labeling of PPIs within ∼ seconds in both in vitro and in vivo systems.^26,32^ Thus, tagging with peroxidases is, in principle, well-suited for tagging proteins associated with the highly dynamic TCR forces.

Herein, we developed a mechanically selective proximity labeling technique, named mechano-ID (Figure 1). Briefly, we used a DNA tension probe composed of three oligonucleotides: (i) a Cy3B fluorescently-labeled ligand strand, (ii) a BHQ2 quencher-modified anchor strand, and (iii) a force-detecting hairpin strand with arms complementary to the ligand and anchor oligos. These three strands fold into a DNA structure that conceals a binding site for a complementary oligonucleotide (lock strand) conjugated to the proximity-tagging enzyme. Thus, the tagging enzyme conjugated to a lock strand is recruited to the concealed binding site only when specific threshold forces unfold the DNA hairpin. Upon the addition of the labeling substrate, the T cells with mechanically active TCRs, as well as the proximal proteins to these TCRs, are tagged by the reactive radicals and can be further collected and analyzed downstream with flow cytometry, proteomics, and Western blot.

**Figure 1:**
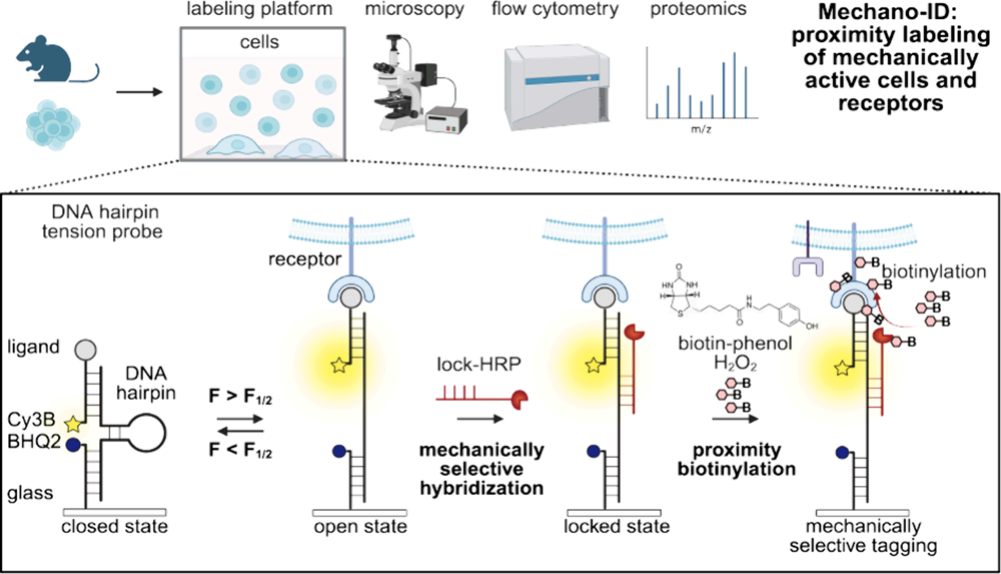
General scheme of mechano-ID: selective proximity biotinylation of mechanically active cells and receptors. Refer to the Methods section in the Supporting Information for a detailed description of surface functionalization (Figure S1). Using immune cells as a model, CD8^+^ T cells were seeded on a glass slide functionalized with DNA tension probes presenting antigens or antibodies. Lock-HRP selectively binds to mechanically unfolded DNA hairpins. Then, when supplied with H_2_O_2_ and biotin-phenol, the membrane proteins in the proximity of TCR forces were covalently tagged with biotin groups.

## Results and Discussion

To demonstrate mechano-ID, we selected horseradish peroxidase (HRP) as the proximity labeling enzyme for four reasons: (a) it has a short labeling time (<1 min); (b) it is commercially available; (c) it is more suitable for extracellular or oxidizing environments;^33,34^ (d) it is amenable to conjugation to other biomolecules such as antibodies to study PPIs.^31^ While Turbo-ID (an improvement to Bio-ID) is another viable enzyme for proximity tagging (labeling time ∼10 min), it is still less efficient than HRP (labeling time ∼1 min).^35^ We first tested whether the conjugation of HRP to the locking oligonucleotide would hinder hybridization (Figure S2). DNA tension probe substrates were prepared as described in Figure S1, and a 17mer complement conjugated to HRP (Figure S3) was added to the solution. We found that hybridization kinetics were not impacted by linking the complement to HRP. Moreover, hybridization was highly sequence-specific, as a scrambled sequence showed minimal binding (Figure S2).

We next tested whether the HRP-DNA conjugates can be recruited selectively to the sites of TCRs that apply threshold forces to the tension probes (Figure 2A). Unless otherwise stated, DNA tension probes were tethered to 8.8 nm gold nanoparticles immobilized onto glass surfaces (Figure S1). In these experiments, TCR forces applied to the ligand exceeding the *F*_1/2_ (the force that leads to a 50% probability of unfolding at equilibrium) unfold the hairpin and expose a cryptic hybridization site. We add a complementary oligonucleotide or lock strand that is conjugated to HRP, delivering the enzyme to mechanically active TCRs. Naïve CD8^+^ T cells from the OT-1 transgenic mice model were used in these experiments since they specifically recognize an 8mer peptide epitope derived from ovalbumin, SIINFEKL, that is bound to MHC. The cells were seeded onto a surface presenting 4.7 pN DNA tension probes linked to antiCD3ε, and the lock-HRP was labeled with Alexa647 (Figure S3) to investigate if hairpin opening and lock-HRP colocalize (Figure 2B). Note that all DNA sequences are listed in Table S1 and were validated in prior work.^5,11,36–39^ 100 nM lock-HRP-647 was added at *t* = 20 min after cell seeding, and RICM and fluorescence images were collected 10 min later to detect cell adhesion and tension, respectively. Note that we maintained a low Alexa647:HRP labeling ratio for these measurements to minimize disruption to HRP catalytic activity, but this generally led to a low fluorescence signal in the Alexa647 channel. We found that the lock-HRP-647 signal overlapped with the tension signal in Cy3B, showing strong colocalization (Figure 2B,C). This confirms that the lock-HRP specifically binds to mechanically unfolded hairpins, in agreement with precedent.^11,37^ Differences between the hairpin opening channel and the lock channel can be ascribed to the fact that DNA hairpin opening is dynamic while lock binding is irreversible at these time scales. To further validate that the lock-HRP conjugation does not hinder cell adhesion or lock binding, we performed experiments quantifying the cell spreading area and the number of opened and locked hairpins as a function of using lock-HRP conjugates compared to a binary mixture of lock and HRP at equal concentrations (lock & HRP) (Figure 2D,E). No statistical difference was found between these groups after 5 min of incubation.

**Figure 2:**
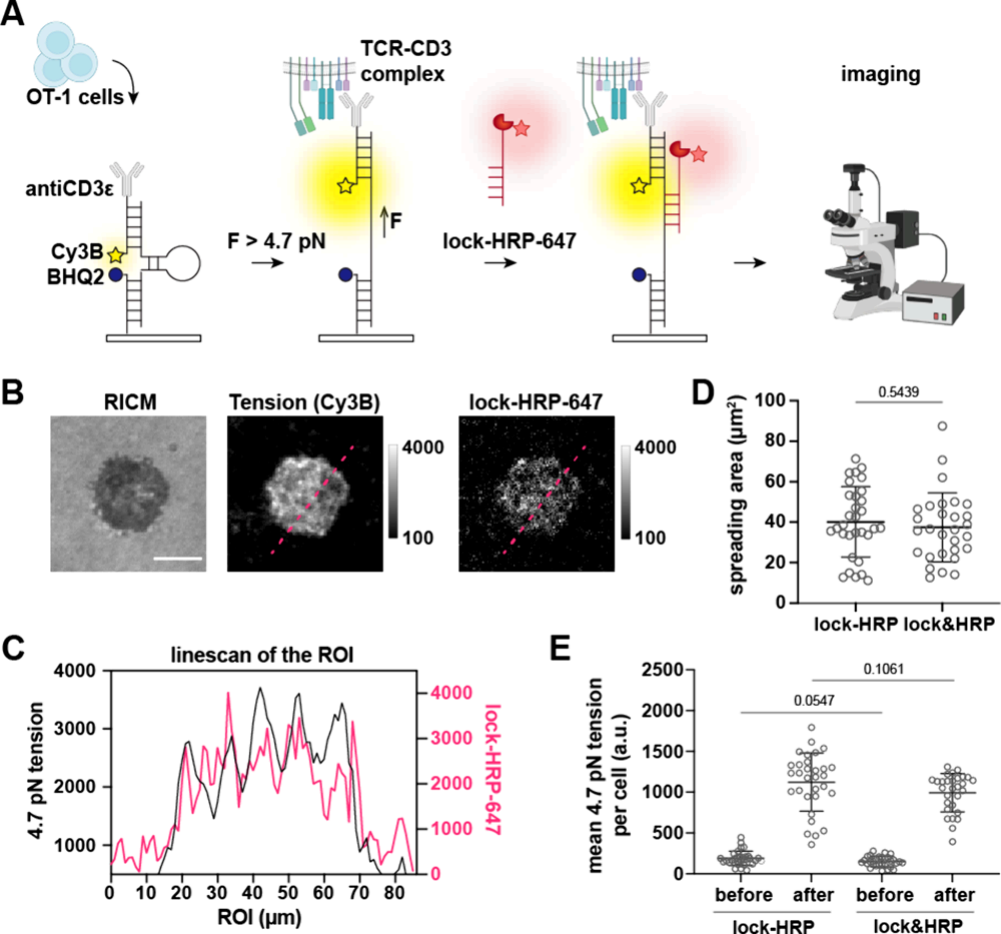
HRP conjugation to lock does not hinder hybridization to mechanically unfolded hairpins. (A) Schematic of the experiments used to measure the mechanically selective hybridization with lock-HRP-647. (B) OT-1 T cells were seeded on antiCD3ε tension probe surface for 20 min followed by addition of lock-HRP-647. Images show representative reflection interference contrast microscopy (RICM), Cy3B tension signal (yellow), and lock-HRP-647 signal (red) acquired 10 min after the addition of 100 nM lock-HRP-647. Scale bar = 5 μm. (C) Linescan of the ROI (raw data) shows the overlapping fluorescence tension signal and lock-HRP-647 signal. (D, E) Quantitative analysis of (D) the spreading area and (E) tension signal before and after mechanically selective hybridization with 250 nM lock-HRP or lock&HRP. Lock&HRP is an equimolar mix of unconjugated lock and HRP, which serves as a control group. *n* = 32 and 29 cells, mean ± SD, unpaired *t* test. Experiment was repeated in triplicate.

Following the validation of mechanically selective hybridization of lock-HRP, we next tested mechanically induced tagging by adding 250 μM biotin-phenol and 1 mM H_2_O_2_ to the well for 1 min and then rapidly quenched with sodium ascorbate (10 mM). The cells were rinsed with HBSS, fixed, and stained with Alexa 488 labeled streptavidin (SA488) to detect proteins in proximity to TCR mechanical events (Figure 3A). Widefield fluorescence microscopy showed a strong SA488 signal that colocalized with the tension signal, as shown in the line scan analysis (Figure 3B,C).

**Figure 3:**
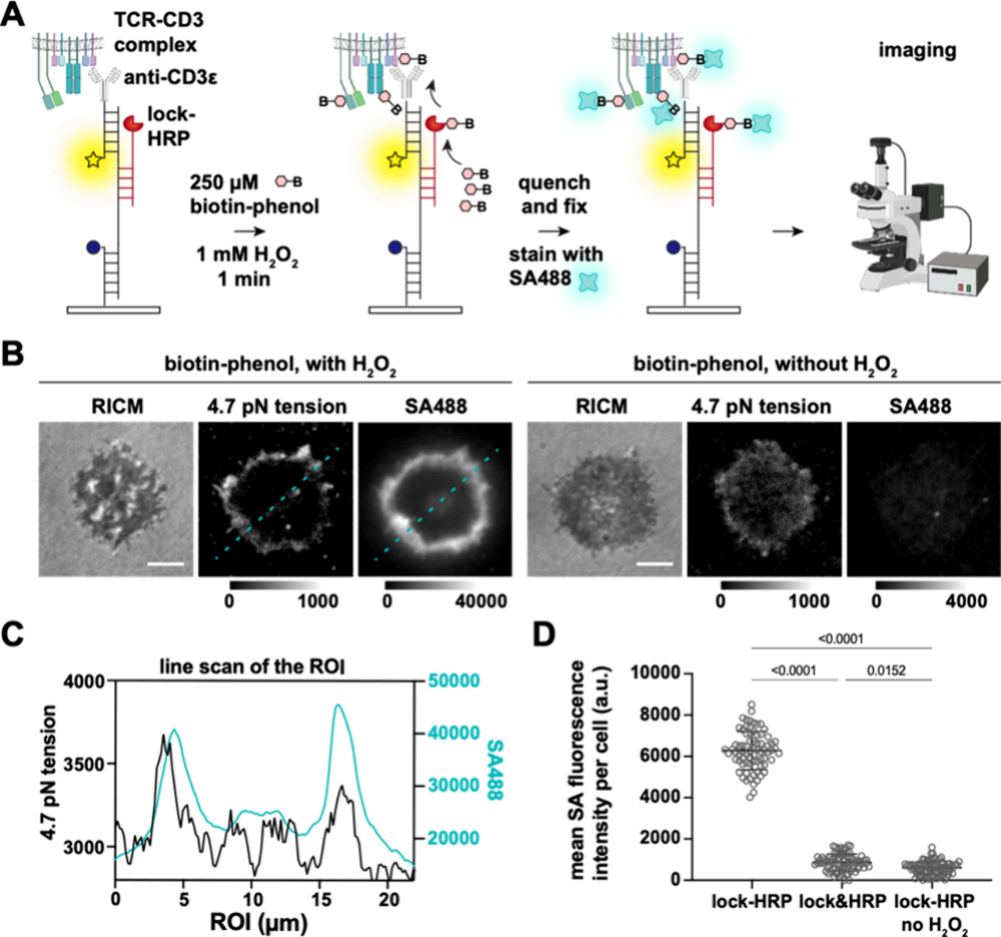
Microscopy analysis of mechanically selective proximity biotinylation. (A) Schematic of experimental design. (B) RICM and fluorescence images of mechano-ID tagged T cells. OT-1 T cells were plated on DNA tension probes that presented antiCD3ε, after the real-time tension signal in the Cy3B channel was observed, lock-HRP was added at 100 nM for 10 min, followed by gentle rinses and proximity labeling. The tagged cell membrane proteins were detected by SA488 after fixing. A negative control without H_2_O_2_ was included. Scale bar = 5 μm. (C) Linescan of the ROI (raw data) shows the overlapping fluorescence tension signal and SA488 signal. (D) Quantitative analysis of the mean SA fluorescence signal per cell after biotinylation. *N* = 74, 80, and 86 cells, mean ± SD. Experiment was repeated in triplicate. Statistical analysis in this figure was performed using an unpaired *t* test.

Colocalization indicates that TCR forces that unfold the 4.7 pN DNA hairpin are in the proximity of sites of biotinylation. It is important to emphasize that TCR forces and hairpin opening are dynamic processes, while HRP-mediated biotinylation only captures longer-lived forces that sustain lock binding and proximity tagging within a specific time window. Negative controls, where H_2_O_2_ was withheld or where the lock and HRP were not conjugated (lock&HRP), showed hairpin opening (Figure S4) but minimal SA488 binding, demonstrating little nonspecific binding and uptake of biotin-phenol and SA488 (Figure 3B,D). Mechanically mediated proximity tagging using lock-HRP on scrambled hairpin probes also showed minimal background tagging (Figure S4). To further validate that tagging is force-mediated, we repeated the experiment using DNA hairpin probes with a greater force threshold of 17 pN, which showed significantly less real-time tension signal and streptavidin staining (Figure S5), in agreement with literature precedent.^37^ Notably, we also observed a heterogeneous level of tension signals even within a monoclonal set of CD8^**+**^ OT-1 T cells (Figure S6). ∼50–60% of the cells showed tension signals on the periphery (Figure 3B), while the remaining cells displayed a more distributed signal (Figure 2B).

We next aimed to read out the mechano-ID signal using flow cytometry analysis. One challenge in establishing the workflow for tagged cell-detection with flow cytometry was harvesting the cells from the DNA tension probe substrate. Here, we utilized the maleimide–thiol DNA tension probes (Figure S1) to avoid release of the nanoparticles, which could increase the background signal. We found that DNase treatment failed to release cells efficiently (Figure S7), potentially a result of DNA modifications due to reactions with phenoxyl radicals.^30^ Therefore, we harvested the cells by gently scraping them off of the glass slides after quenching the labeling solution. After performing proximity biotinylation on the substrate, OT-1 T cells were harvested and washed. They were then incubated on ice with Alexa647 labeled streptavidin (SA647) for 10 min, after which they were washed again and ran through a flow cytometer (Figure 4A). The OT-1 T cells were allowed to mechanically interact with substrates presenting either antiCD3ε, their cognate antigen pMHC N4 (SIINFEKL), or an altered peptide ligand, pMHC Q4 (SIIQFEKL). As the binding of CD8 to the MHC was identified as a main contributor to the TCR forces, we tested whether there would be less labeling if CD8-MHC binding was blocked using a CD8 blocking antibody (clone CTCD8a). Cells incubated with the nonlinked lock&HRP were used as a negative control for nonspecific proximity tagging. Though the highest real-time tension signal was typically observed for cells seeded on the antiCD3ε probes, the pMHC N4 presenting probes yielded the greatest locked tension signal (Figure 4B). This is consistent with our prior work showing that the locked tension signal accumulated at a faster rate for antigens due to frequent and repeated mechanical sampling with forces exceeding 4.7 pN (Figure 4B).^11^ As expected, pMHC Q4 and blocked CD8 samples showed weak locked tension signals in microscopy (Figure 4B and S8), whereas conjugation of the lock to HRP did not alter the accumulated lock signal for pMHC N4 (no notable differences between lock-HRP and lock&HRP).

**Figure 4:**
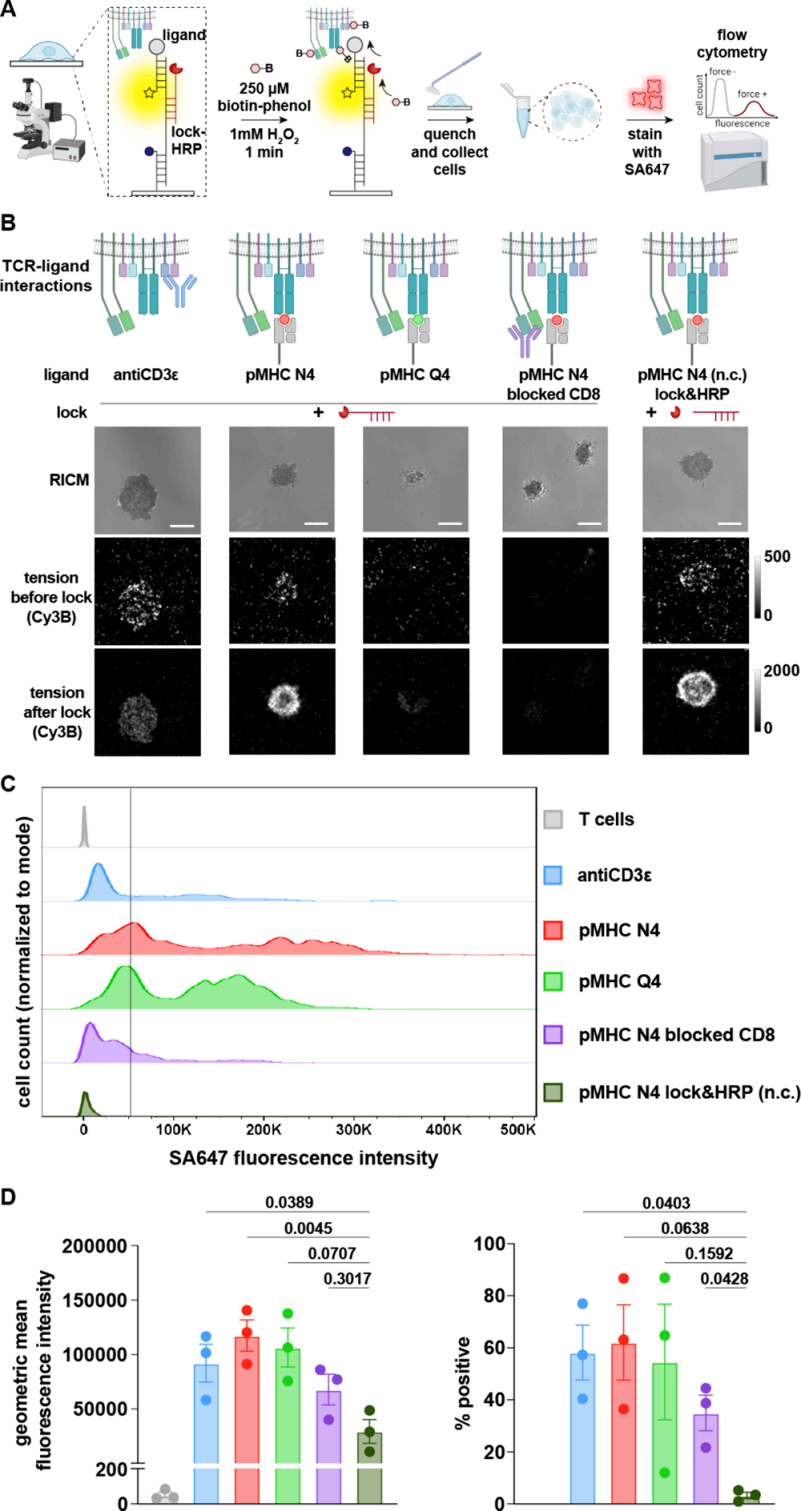
Flow cytometry analysis of mechano-ID tagged OT-1 T cells challenged with different ligands. (A) Schematic showing the workflow. (B) Microscopy images of OT-1 T cells spread on DNA tension probe substrates presenting antiCD3ε, pMHC N4, and pMHC Q4. Real-time Cy3B signals of TCR forces were acquired, followed by the addition of 250 nM lock-HRP and image acquisition of the accumulated tension signal in the Cy3B channel after 5 min. The T cells with blocked CD8 and the T cells on pMHC N4 substrate incubated with lock&HRP were included as controls. Uncontrasted microscopy images for blocked pMHC Q4 and CD8 are found in Figure S8. Scale bar = 5 μm. (C) Representative flow cytometry detection of the OT-1 T cells with mechanically active TCRs. After image acquisition, proximity biotinylation was performed, after which the cells were rinsed, collected, and stained with SA647. Three biological replicates were performed with each generating consistent data. The gating strategy is shown in Figure S9. (D) Average geometric mean fluorescence intensity of the cells and % positive of the cells from 3 biological replicates. Error bars represent SD. Statistical analysis was performed using one-way ANOVA and multiple comparison with lock&HRP control.

The flow cytometry results were in agreement with the representative microscopy images for pMHC N4 and antiCD3ε (Figure 4C). Interestingly, the pMHC Q4 showed a similar level of signal in flow to pMHC N4, which did not match the intensity in microscopy. This may be due to the nonlinear amplification signals of mechano-ID. The fluorescent microscopy images reflect inherent differences in force kinetics while the fluorescent signals in flow showed the amplification effect of mechano-ID labeling. While TCRs engage N4 more frequently than Q4, the amplification effect of peroxidase obscures the difference by saturating the dynamic range of detection, resulting in plateaus in proximal tyrosine labeling. When CD8-MHC binding was blocked, the level of mechano-ID tagging was notably reduced, in agreement with the previous report on the TCR force with CD8 blocking.^10^ Overall, the labeling levels across the different treatment groups showed strong correlation with the TCR-pMHC mechanics reported previously.^11^ Moreover, the percentage of cells that showed positive biotinylation in each sample also agreed with the potency of the TCR interaction with pMHC.^40^ We noticed highly heterogeneous levels of mechanical labeling in the flow cytometry readout (Figure 4C,D), agreeing with the heterogeneous distribution in mechanical activity observed in microscopy data (Figure S6). It is important to note that proximity tagging will also lead to self-tagging of HRP and hence some of the streptavidin staining observed in microscopy (Figure 3) is not necessarily on the cell membrane. However, flow cytometry, in contrast to microscopy, measures biotinylation on the cell membrane and is a more faithful measure of the bona fide mechanical tagging of target receptors and cells.

Next, we performed proteomic analysis to identify proteins tagged using mechano-ID. Here, we used both antiCD3ε and pMHC N4 mechano-ID probes, and as controls, we repeated an identical procedure but using HRP that was not conjugated to the lock (lock&HRP) mirroring the controls for the flow and microscopy experiments. In this case, we employed the DBCO-azide conjugation of DNA tension probes (Figure S1) onto the surface, because this strategy yielded a uniform high yield protein isolation. Following cell labeling with the various mechano-ID probes, biotinylated proteins were enriched using streptavidin beads and subsequently analyzed by mass-spectrometry-based proteomics (Figure 5A). But in contrast to experiments for microscopy and flow cytometry, we used large numbers of cells (10 × 10^6^ cells per experiment). We first compared the enrichment of TCR-CD3 complex members and canonical proteins known to be recruited upon TCR binding (Figure 5B). We found significant enrichment of TCRα, TCRβ, CD3γ, CD3δ, CD3ε, CD8α, and CD8β compared to the controls (Figure 5C). We also performed Western blotting to validate our proteomic findings. Staining with HRP-conjugated anti-TCRα (H28) showed a band only for lock-HRP but not for lock&HRP (Figure S10), which is in agreement with our proteomics analysis (Figure S11). Importantly, intracellular proteins associated with the TCR such as Zap70, Lck, and Plcγ1 were not enriched upon mechano-ID labeling (Figure 5C) since biotin phenoxyl radicals are not membrane permeable.^34,41^ Proteins with small extracellular domains showed weak (CD3ζ) or nonsignificant (Lat) enrichment given that aromatic residues in the extracellular domain are the primary target for mechano-ID labeling. For example, CD3ζ has a single aromatic residue^42^ while Lat has none in the extracellular domain.^43,44^

**Figure 5:**
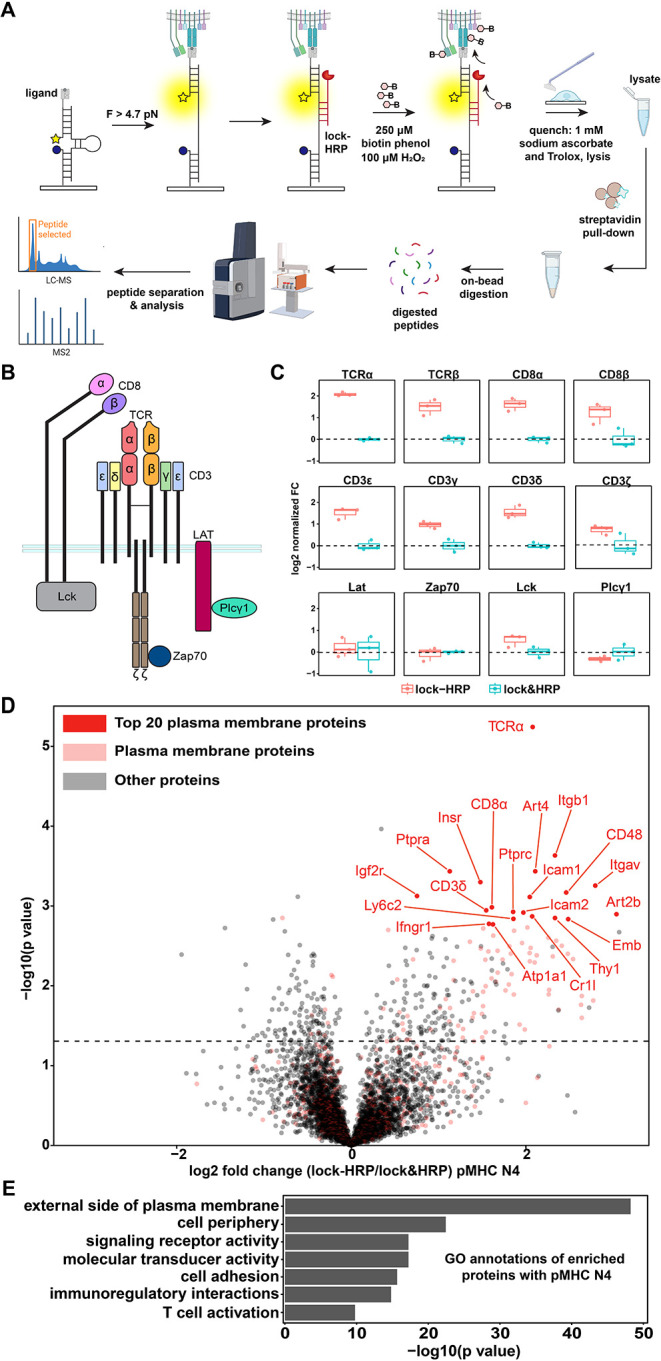
Proteomic profiling of enriched proteins after mechano-ID. (A) Workflow for proteomic analysis to obtain labeled mechanically active proteins. (B) Illustration of known proteins associated with TCR engagement. (C) Box plots of 12 known proteins to directly associate with TCR engagement using mechano-ID with pMHC N4 probe. FC= fold change, red and blue represent the positive lock-HRP and negative lock&HRP groups, respectively. Fold change and *p* values are shown in Table S4. Experiment was performed in triplicate. (D) Volcano plot showing differentially enriched ectodomain proteins labeled using mechano-ID with pMHC N4 probe. Ectodomain proteins are highlighted in red while all other proteins are shown in gray. The *y*-axis indicates statistical significance while *x*-axis indicates the FC between positive and negative groups. *p* value <0.05 and FC > 0.75. (E) Gene ontology annotations of significantly enriched proteins by mechano-ID with pMHC N4.

We conducted an unbiased statistical analysis comparing the pMHC N4 mechano-ID probes. Among the top 20 enriched ectodomain proteins, several were associated with key immune functions, including adhesion and migration (Icam1, Icam2, Itgav, Itgb1, Emb, Ptpra, Cr1l), T cell activation (CD48, Ptprc, CD8α, CD3δ, Ifngr1), and signal transduction (TCRα, Insr, Atp1a1, Ly6c2, Thy1) (Figure 5D). These functional associations were further supported by gene ontology (GO) enrichment analysis (Figure 5E). We also observed a strong overlap in the protein enrichment profiles between pMHC N4 and antiCD3ε mechano-ID probes, with 39 proteins significantly enriched by both ligands (Figure S12). Of these 39 proteins, 18% are integrin subunits (Table S5). Integrins are mechanosensors, and this data indicates the proximity of integrins to TCR forces and suggests that integrins are recruited and involved in the initial TCR triggering. >90% of these 39 similar proteins are functionally linked to cell adhesion and migration, immunoregulation, and T cell activation processes.

## Conclusions

Compared to affinity and avidity-based techniques to characterize antigen-TCR potency, such as tetramer staining and surface plasmon resonance (SPR),^16^ TCR forces offer a complementary functional marker for immunogenicity.^45^ Current microscopy-based techniques to detect TCR-antigen forces have limited throughput in identifying mechanically active T cells and TCRs. Therefore, we sought to develop a method that can identify and isolate T cells with mechanically active TCR-pMHC bonds. Mechano-ID offers a platform technology that can selectively label mechanically active TCR-ligand interactions by biotinylating these receptors and their proximal proteins. The signal generated by mechano-ID can be detected by using flow cytometry or by protein analysis, including proteomics and Western blotting. The signal intensity quantified by microscopy and flow cytometry corresponds to the frequency and duration of mechanical engagement between the TCR and its antigens, and this signal intensity is a potential marker of ligand activity.

One important limitation of mechano-ID, in its current form, is that the forces need to be below ∼20 pN, which is the threshold of force-induced peeling, as that would release the enzyme-labeled strand or hinder its binding.^46^ Moreover, in the current workflow, there is a time delay (5–10 min) between the mechanical event and the chemical tagging step, which may lead to tagging of mechanical history rather than real-time mechanical events. However, this issue may be overcome with microfluidics to accelerate the mixing and washing.

Our observations in flow and microscopy showed that transgenic T cells derived from a single animal display heterogeneous levels of mechanical activity. This raises interesting questions, for example: what contributes to the dramatic differences in T cell mechanical responses to the same antigen? With this tool at hand, it may be possible to pursue the next step of sorting mechanically active T cells and attributing differences in force with RNA and protein expression levels.^47–49^ In principle, mechano-ID may also allow the labeling of mechanically active TCRs in polyclonal populations to identify potent clones with proteomics. Although we have not confirmed this, it is plausible that the mechanically active antigens are also biotinylated, which provides a powerful approach to identify potent TCR-pMHC pairs using a pMHC library to challenge polyclonal T cell samples. Therefore, mechano-ID may enable using force to predict T cell responses, as well as identifying antigens for personalized cancer immunotherapies.^50^ Moreover, recent reports expanding the proximity labeling toolbox using photocatalytic approaches^51–55^ are likely amenable to integration with mechano-ID. Finally, we would like to emphasize that mechano-ID is a platform technology that can be used to study a variety of mechanotransduction pathways including Notch/Delta,^56^ cadherins,^57^ and others.

## Supplementary Material



## Notes

Supporting Information contains detailed descriptions of materials and methods for the tool development, as well as supporting data (PDF)

## †

Department of Bioengineering, Stanford University, 443 Via Ortega, Stanford, California 94305, United States

## Abbreviations

TCR: T cell receptor
pMHC: peptide major-histocompatibility complex
HRP: horseradish peroxidase
APEX: ascorbate peroxidase
SA: streptavidin
