# Supplementary material for: Mechano-ID: Proximity Labeling of Mechanically Active Receptors Reveals the Mechanome and Tags Mechanically Active Cells
Source: J Am Chem Soc. 2025 Sep 26;147(40):36097–104. doi: 10.1021/jacs.5c05203 (PMC12512101; doi:10.1021/jacs.5c05203)
Supplement: Supplementary file 1 [file ja5c05203_si_001.pdf]

Supporting information for

Mechano-ID: Proximity Labeling of Mechanically Active Receptors Reveals the Mechanome and Tags Mechanically Active Cells

Rong Ma[1‡†], Mohamed Husaini Bin Abdul Rahman[1‡], Christian M. Beusch[3,4], Brendan R. Deal[1], David E. Gordon[3], Khalid Salaita[1,2*]

[1] Department of Chemistry, Emory University, 1515 Dickey Drive, Atlanta, GA 30322, United States

[2] Wallace H. Coulter Department of Biomedical Engineering, Emory University and Georgia Institute of Technology, 313 Ferst Drive NW, Atlanta, GA 30332, United States

[3] Pathology Advanced Translational Research Unit (PATRU), Department of Pathology and Laboratory Medicine, Emory University, 1462 Clifton Road, Atlanta, GA 30322, United States

[4] Department of Surgical Sciences, Uppsala University, Dag Hammarskjölds väg 7, 752 37 UPPSALA, Sweden

* Corresponding author email: k.salaita@emory.edu

‡ *R.M* and *M.H.B.A.R* contributed equally to this work.

Materials and methods

1. Materials

The oligonucleotides used in this study are listed in **Table S1.** The reagents used in this study are listed in **Table S2.** And the equipment used in this study is listed in **Table S3.**

Table S1. List of oligonucleotides.

Oligo name	5’	Sequence (5’ to 3’)	3’
Amine ligand strand (A21B)	/5AmMC6/	CGC ATC TGT GCG GTA TTT CAC TTT	/3Bio/
Cy3B ligand strand (A21b Cy3B)	Cy3B	CGC ATC TGT GCG GTA TTT CAC TTT	/3Bio/
BHQ2 anchor strand	/5ThiolMC6-D/	TTT GCT GGG CTA CGT GGC GCT CTT	/3BHQ_2/
4.7 pN hairpin strand	-	GTG AAA TAC CGC ACA GAT GCG TTT GTA TAA ATG TTT TTT TCA TTT ATA CTT TAA GAG CGC CAC GTA GCC CAG C	-
4.7 pN hairpin 15mer lock	-	AAA AAA CAT TTA TAC	-
4.7 pN hairpin 15mer lock amine	-	AAA AAA CAT TTA TAC CCT ACC TA	/3AmMO/
4.7 pN hairpin 15mer lock thiol	-	AAA AAA CAT TTA TAC CCT ACC TA	/3ThioMC3-D/
4.7 pN hairpin 15mer lock-HRP	-	AAA AAA CAT TTA TAC CCT ACC TA	HRP
Scrambled 4.7 pN hairpin strand	-	GTG AAA TAC CGC ACA GAT GCG TTT GTA AAT ATG TGG TGG TCA TAT TTA CTT TAA GAG CGC CAC GTA GCC CAG C	-
4.7 pN hairpin 17mer lock	-	GAA AAA AAC ATT TAT AC	-
4.7 pN hairpin 17mer lock thiol	-	GAA AAA AAC ATT TAT ACC CTA	/3ThioMC-D/
4.7 pN hairpin 17mer lock-HRP	-	GAA AAA AAC ATT TAT ACC CTA	HRP
BHQ2 anchor strand with DBCO	/5DBCON/	TTT GCT GGG CTA CGT GGC GCT CTT	/3BHQ_2/
17 pN hairpin strand	-	GTG AAA TAC CGC ACA GAT GCG TTT CGG GCC GGC GCG CGG ATT AAT CCG CGC GCC GGC CCG TTT AAG AGC GCC ACG TAG CCC AGC	-
17 pN 22mer lock	-	GAT TAA TCC GCG CGC CGG CCC GCC TAC CTA	-
17 pN hairpin 22mer lock thiol	-	GAT TAA TCC GCG CGC CGG CCC GCC TAC CTA	/3ThioMC3-D/
17 pN hairpin 22mer lock-HRP	-	GAT TAA TCC GCG CGC CGG CCC GCC TAC CTA	HRP

Table S2. List of reagents.

Name of material/item	Company	Catalog Number
Biotin anti-mouse CD3ε Antibody (2C11)	Biolegend	100304
Biotinylated pMHC ovalbumin (SIINFEKL)	NIH Tetramer Core Facility	NA
Biotinylated pMHC ovalbumin (SIIQFEKL)	NIH Tetramer Core Facility	NA
Anti-Mouse CD8a (Ly 2) Purified (Clone CT-CD8a) (rat IgG2a)	Cedarlane	CL168AP
10x Red blood cell lysis buffer	Biolegend	00-4333-57
Dulbecco's phosphate-buffered saline (DPBS)	Corning	21-031-CM
Hank's balanced salts (HBSS)	Sigma	H8264
BD Syringes only with Luer-Lok	BD Bioscience	309657
Cell strainers	Biologix	15-1100
Midi MACS (LS) startup kit	Miltenyi Biotec	130-042-301
Mouse $CD8^+$ T cell isolation kit	Miltenyi Biotec	130-104-075
Ovalbumin (257-264) chicken	Sigma	S7951-1MG
Atto647N NHS ester	Sigma	18373-1MG-F
Alexa Fluor™ 488 NHS ester	Thermo Fisher	A20100
Alexa Fluor™ 647 NHS ester	Thermo Fisher	A20006
Cy3B NHS ester	GE Healthcare	PA63101
3-Hydroxypicolinic acid (3-HPA)	Sigma	56197
Nanosep MF centrifugal devices	Pall Laboratory	ODM02C35
Bio-gel P2 gel extra fine polyacrylamide beads	Bio-rad	1504118
Triethylammonium acetate buffer	Sigma	90358
mPEG-SC	Biochempeg	MF001023-2K
(3-Aminopropyl)triethoxysilane	Acros	AC430941000
8.8 nm gold nanoparticles, tannic acid	Nanocomposix	Customized order
Coverslip Mini-Rack, Teflon	Thermo Fisher Scientific	C14784
Ethanol	Sigma	459836
Hydrogen peroxide	Sigma	H1009
LA-PEG-SC	Biochempeg	HE039023-3.4K
Sulfuric acid	EMD Millipore Corporation	SX1244-6
SMCC (succcinimidyl 4-(N-maleimidomethyl)cyclohexane-1-carboxylate	Thermo Fisher	22360
Sulfo-NHS acetate	Thermo Fisher Scientific	26777
Wash-N-Dry™ Slide Rack	Sigma	Z758108
Glass Coverslips for sticky- Slides 25 / 75 mm	Ibidi	10812
Sticky-slide 18 Well	Ibidi	81818
Bovine serum albumin	Sigma	735078001
Attofluor Cell Chamber, for microscopy	Thermo Fisher Scientific	A7816
Dimethyl Sulfoxide (DMSO)	EMD Millipore Corporation	M1096780100
Sodium azide	Sigma	S2002
UltraPure™ 0.5M EDTA, pH 8.0	Thermo Fisher	15575020
Formaldehyde solution	Sigma	252549
Triton™ X-100	Sigma	X100
Sulfo-SMCC (sulfosucccinimidyl 4-(N-maleimidomethyl)cyclohexane-1-carboxylate	Thermo Fisher	22322
Streptavidin	Thermo Fisher	434302
Biotinyl tyramide	Sigma	SML2135
Sodium ascorbate	Sigma	Y0000039
Amicon® Ultra 0.5 mL Centrifugal Filters	Sigma	UFC503024
Horseradish peroxidase (HRP)	Sigma	P8250
Bond-Breaker™ TCEP Solution, Neutral pH	Thermo Fisher	77720
Trolox®, 97%	Thermo Fisher Scientific	218940050

Azido-PEG4-NHS ester	BroadPharm	BP-20518
Bovine Serum Albumin (BSA) Fraction V	Sigma	10735078001
TCR alpha antibody (H28-710) with HRP	Santa Cruz Biotechnology	Sc-101410HRP
TCR beta antibody (H57-597)	Biolegend	109201
ProPlate® Spring Clip- Tray & Cover	Grace Bio-labs	246879
ProPlate® Stainless Steel Spring Clips	Grace Bio-labs	204838
ProPlate® Multi-Array Slide System- 1 Well Slide Module	Grace Bio-labs	246851
Trypsin/Lys-C Mix, Mass Spec Grade	Promega	V5073
Hank's balanced salts (HBSS)	Sigma	H6648
Sodium bicarbonate	Fisher Chemical	S233-500
Bio-gel P4 gel extra fine polyacrylamide beads	Bio-rad	1504128
Parafilm	Amcor	PM996
Sodium Chloride	Aqua Solutions	S2675-500G
Proplate® 96 Round Well, Bottomless adhesive microtiter plate	Grace Bio-labs	204969
Pierce® RIPA buffer	Thermo Fisher Scientific	89900
Phenylmethylsulfonyl fluoride (PMSF)	Thermo Fisher Scientific	36978
HALT™ Protease inhibitor cocktail (100X)	Thermo Fisher Scientific	78429
Cell culture scraper	Biologix	70-1180
Micro BCA™ Protein Assay kit	Thermo Fisher Scientific	23235
Pierce® Streptavidin magnetic beads	Thermo Fisher Scientific	88817
4X Laemmli buffer	Bio-rad	1610747
B-mercaptoethanol	Sigma	M7522-100ML
LF PVDF membrane	Bio-rad	1620261
Trans-Blot Turbo Mini Filter Paper	Bio-rad	12023835
Trans-Blot Turbo 5X Transfer buffer	Bio-rad	10026938
10X Tris Buffered Saline (TBS)	Bio-rad	1706435
10X Tris/Glycine/SDS	Bio-rad	1610732
Clarity Western ECL substrate	Bio-rad	1705061
Tween-20	Fisher Scientific	BP337-500
Urea	Fisher Scientific	AA3642822
Tris-HCl (1 M), pH 8.0	Fisher Scientific	AM9855G
n-Dodecyl-b-D-maltoside (DDM)	Thermo Fisher Scientific	89903
Chloroacetamide	VWR	TCC0086-025G
Trifluoroacetic acid	VWR	BJ302031-100ML
Evotip Pure	Evosep	EV2013

Table S3. List of equipment.

Equipment	Company
Barnstead Nanopure water purifying system	Thermo Fisher
AdvanceBio Oligonucleotide C18 column, 4.6 ×150 mm, 2.7 µm	Agilent
High-performance liquid chromatography	Agilent
Matrix-assisted laser desorption/ionization time-of-flight mass spectrometer (MALDI-TOF-MS)	Voyager STR
Nanodrop 2000 UV-Vis Spectrophotometer	Thermo Fisher
CFI60 Apochromat TIRF 100x Oil Immersion Objective Lens, N.A. 1.49	Nikon
Prime 95B-25MM Back-illuminated sCMOS Camera. 1608 ×1608, 30fps	Photometrics
Nikon Ti2-E Motorized Research Microscope	Nikon
Ti2-ND-P Perfect Focus System 4	Nikon
SOLA SE II 365 Light Engine	Nikon
NIS Elements software	Nikon
C-FL Surface Reflection Interference Contrast (SRIC) Cube	Chroma Technology
CF-L AT CY5/Alexa Fluor 647/Draq 5 Filter Set	Chroma Technology
C-FL DS Red Hard Coat, High Signal-to-Noise, Zero Shift Filter Set	Chroma Technology
CytoFLEX V0-B3-R1 Flow Cytometer	Beckman Coulter
Bio WAX, NP5, SS, 5 um, non-porous, 4.6 ×250 mm	Agilent
iBright FL1500 Imaging System	Thermo Fisher
timsTOF Pro 2 mass spectrometer	Bruker Daltonics
Evosep One	Evosep
Aurora Elite™ 15×150 CSI C18 UHPLC column	Ionopticks
T100 Thermal Cycler	Bio-rad

2. Methods

Mice and cells

Briefly, OT-1 transgenic mice were housed and bred in the Division of Animal Resources Facility at Emory University under the Institutional Animal Care and Use Committee. OT-1 T cells that express the CD8 co-receptor and specifically recognize chicken ovalbumin epitope 257–264 (SIINFEKL) were isolated and enriched from the spleen of a sacrificed mouse using MACS system and $CD8^+$ T cell isolation kit according to the manufacturer's instruction. DPBS buffer supplemented with 0.5% BSA and 2 mM EDTA was used for the purification process as described in the manufacturer's instruction. The purified $CD8^+$ naïve OT-1 cells were kept in HBSS at 2×10^6 cells/mL on ice before imaging. These were used the same day after purification.

Oligonucleotide preparation

Oligo-dye conjugation. Cy3B ligand strand and lock-647N were prepared by N-hydroxysuccinimide (NHS) reaction. Briefly, 50 µg (excess amount) of NHS dye was dissolved in 10 µL of DMSO and reacted with 10 nmol of oligonucleotide in 1×PBS containing 0.1 M $NaHCO_3$ overnight at 4 °C or 1 h at room temperature (**Figure S3A**). After the reaction, byproducts, salts, and unreacted dye in the mixture were removed by P2 gel filtration using Nanosep MF centrifugal devices. The product was further purified by reverse-phase HPLC equipped with Agilent AdvanceBio Oligonucleotide C18 column (653950-702, 4.6 ×150 mm, 2.7 µm). The mobile phase A: 0.1 M TEAA and B: ACN were used for a linear gradient elution of 10-100% B over 50 min at a flow rate of 0.5 mL/min (**Figure S3**). The desired product was characterized by MALDI-TOF-MS (data not shown) and the concentration of the oligo-dye is determined by UV-Vis using absorbance at 260 nm (data not shown).

Lock-HRP conjugation. Lock-HRP was prepared by conjugating the thiol lock strand with HRP using a heterobifunctional crosslinker, sulfo-SMCC (**Figure S3B**). 44 µL of 10 mg/mL (227 µM) HRP was prepared in 1×PBS (pH 7.2) and added to 44 µL of 2 mg/mL sulfo-SMCC in DI water (4.6 mM) at room temperature for 30 min to 1 h. Excess crosslinker was removed using a desalting column with PBS-hydrated P4 gel twice. Meanwhile, the disulfide group on the thiol lock strand was reduced following the manufacturer's instructions. Briefly 30 nmols of thiol lock strand (30 µL of 1 mM DNA in DI water) was added to 12 µL of 0.5 M TCEP (200× molar excess) at room temperature for 15 min. This reduced thiol strand solution was then mixed with the product of the maleimide-activated HRP, without any dilution, at room temperature and allowed to react for 1 h. The product was purified using ion-exchange column chromatography and validated using a 10% resolving SDS-PAGE gel (**Figure S3**).

For microscopy experiments, the lock-HRP was then labeled with Alexa 647 on remaining lysine residues. To achieve this coupling reaction, we mixed 10 nmol of lock-HRP product with 50 µg Alexa 647 NHS ester dissolved in 10 µL of 1X PBS. The lock-HRP-647 product was purified by P4 gel. The concentrations of the lock-HRP and lock-HRP-647 stocks were characterized using UV-Vis at 260 nm after baseline correction (**Figure S3C**). The ratio of absorbances at 260, 280, 403, and 647 nm provided further validation of the purity of the product. Achieving a lock-HRP-647 conjugate with a 1:1:1 ratio of DNA: HRP:

Alexa647 was difficult because of the availability of multiple lysine residues on the HRP and also because excess unreacted HRP was difficult to fully remove. Nonetheless, achieving parity in the ratio is not required as free HRP is washed away before proximity biotinylation, and the HRP molecule conjugated to more than one lock is unlikely to affect the biotinylation result.

Fluorescence labeling of streptavidin

Streptavidin was labeled with Alexa 488 NHS ester or Alexa 647 NHS ester. Briefly, 100 µg streptavidin was reconstituted in 100 µL 1×PBS and added to a 50 µg dye aliquot. The mixture was allowed to react for 30 min at room temperature. The product was purified with P4 gel filtration hydrated with PBS to remove unreacted dye and by-products. The SA488 and SA647 were characterized using UV-Vis and the concentration was determined by the absorbance at 280 nm.

Surface preparation

Amine modified glass slides (**Figure S1**). Glass slides (25×75 mm) were rinsed with water three times and placed onto a Wet-N-Dry rack in a 200 mL tall beaker. The glass slides were submerged in ethanol and sonicated to clean for 15 min. Then the ethanol was disposed of, and the slides were submerged in water and sonicated to clean for another 15 min. The cleaned glass slides were washed with water 6 times to remove any residual ethanol and dust. Piranha solution (200 mL) was prepared in a clean 200 mL beaker by mixing sulfuric acid and H_2O_2 at a ratio of 3:1 v/v. (CAUTION: Piranha solution is highly reactive and hazardous. It may explode if H_2O_2 exceeds 50% or if it is mixed with organic solvents.) After gentle mixing, the rack that held the slides was transferred to the beaker containing the fresh piranha solution and etched for 30 min at room temperature. After etching, the rack that held the glass slides was transferred to a new beaker and rinsed with 200 mL water 6 times, followed by another 3 washes with 200 mL ethanol. APTES solution (200 mL) was then prepared at 3% w/v in ethanol in a 200 mL beaker, and the rack was submerged in the APTES solution and sealed with parafilm to react for 1 h at room temperature. After the reaction, the rack holding the slides was transferred to a new beaker and washed with ethanol 6 times. The slides were then baked dry at 80 °C for 20 min. The amine modified glass slides are stored at -20 °C until use.

There were three main chemistries used to anchor the tension probes to the substrate. All have been reported previously and yield similar high-quality results. Note that the gold nanoparticle tethered probes were used to obtain the data shown in **Figures 2** and **3**. The maleimide-thiol chemistry was used for the flow cytometry data (**Figure 4**) to avoid the potential of the scraping method physically releasing the nanoparticles off the substrate. Finally, DBCO-azide chemistry was used to collect the proteomics and western blotting experiments because this was more convenient to obtain uniform high yield protein isolation which was needed for western blotting.

Gold particle DNA-tension probe substrate (**Figure S1A**). An amine modified glass slide was placed in a petri dish, of which the bottom is covered with parafilm. Lipoic acid-PEG NHS (6 mg) and mPEG NHS (30 mg) were weighed and dissolved in 1.2 mL 0.1 M $NaHCO_3$ immediately before adding to the amine glass slide and allowed to react for 1 h

at room temperature. After the reaction, the glass slide was rinsed with water and incubated with 10 mg/mL sulfo-NHS acetate in 0.1 M $NaHCO_3$ for 30 min at room temperature for passivation, and then washed with water. Afterwards, the lipoic acid PEG functionalized glass slide was air dried and assembled with a sticky-slide chamber. Gold nanoparticles were added to the wells at 0.05 mg/mL (80 µL/well) and allowed to immobilize for 30 min at room temperature in the dark. Meanwhile, DNA tension probes were annealed by heating a 1.1:1:1 mixture of the 4.7 pN hairpin, Cy3B ligand strand, and BHQ2 anchor strand at 300 nM in 1 M NaCl to 95 °C for 5 min, and gradually cooling down to 20 °C over 20 min. After the gold particle immobilization, the unbound particles were washed away with sufficient water. DNA tension probes were mixed with another 9-fold excess BHQ2 anchor strand (total final concentration of BHQ2 anchor strand is 3 µM) after annealing and added to each well (40 µL/well) for overnight incubation. On the second day, the excess unbound DNA probe was washed away with PBS and then streptavidin (40 µg/mL, 50 µL/well) was added to the wells and allowed to incubate for 30 min at room temperature. Each well was then washed with PBS and the biotin-antibody (40 µg/mL, 50 µL/mL) or biotin-ligand (10 µg/mL, 50 µL/well) was added to the surfaces and incubated for 30 min at room temperature. After the immobilization of the ligand, the wells were washed with PBS and were ready for imaging.

Maleimide-thiol DNA-tension probe substrate (**Figure S1B**)**.** An amine modified glass slide was placed in a petri dish, of which the bottom is covered with parafilm. SMCC (4 mg) was weighed and dissolved in 400 µL DMSO immediately before adding to the amine glass slide and allowed to react for 30 min at room temperature. Meanwhile, DNA tension probes were annealed by heating the 1.1:1:1 mixture of the 4.7 pN hairpin, Cy3B ligand strand, and BHQ2 anchor strand at 100 nM in 1×PBS (pH = 6.8) to 95 °C for 5 min, and gradually cooling down to 20 °C in 20 min. After the SMCC reaction, the glass slide was rinsed with ethanol and incubated with 400 µL 10 mg/mL sulfo-NHS acetate in DMSO for 30 min at room temperature for passivation, and then rinsed with ethanol. In the meantime, 200-fold molar excess of TCEP was added to the annealed DNA tension probes for 15 min to reduce the thiol on the BHQ2 anchor strand. After the passivation, the maleimide activated glass slide was air dried and assembled with a sticky-slide chamber. The reduced DNA tension probe (40 µL/well at 100 nM) was added to the wells and incubated for 1 h at room temperature. After the immobilization of the DNA tension probes, the wells were washed with 1×PBS and were further passivated with 0.5% BSA in PBS for 5 min at room temperature. Then, streptavidin (50 µg/mL, 50 µL/well) was added to the wells for 30 min incubation at room temperature, after which the wells were washed with PBS and incubated with biotin-antibody (40 µg/mL, 50 µL/well) or biotin-ligand (10 µg/mL, 50 µL/well) for 30 min at room temperature. After the incubation, the wells were washed with PBS and ready for experiments.

Azide-DBCO DNA-tension probe substrate (**Figure S1B**)**.** The following protocol is similar to the one described above for the SMCC-thiol coupling. Briefly, an amine modified 18 mm by 18 mm glass slide was assembled on adhesive-bottomed 96 well plate. 10 mg/mL NHS-PEG4 azide in 0.1 M sodium bicarbonate solution (50 µL/well) was added and allowed to react for 1 h at room temperature. After the NHS-PEG4-azide reaction, the glass slide was rinsed with nanopure water and incubated with 1 mg/mL sulfo-NHS

acetate in 0.1 M sodium bicarbonate (100 µL/well) for 1 h at room temperature for passivation, and then rinsed with nanopure water. Meanwhile, DNA tension probes were annealed by heating the 1.1:1.1:1 mixture of the 4.7 pN hairpin (or 17 pN hairpin), Cy3B ligand strand, and BHQ2 anchor strand with DBCO at 100 nM in 1 M NaCl to 95 °C for 5 min, and gradually cooling down to 20 °C in 30 min. After sulfo-NHS acetate passivation, the glass slide was passivated with 0.1% BSA in 1×PBS (100 µL/well) for 30 min and rinsed with 1×PBS. The DBCO-functionalized DNA tension probe (50 µL/well at 100 nM) was added to the wells and incubated at room temperature overnight. After the immobilization of the DNA tension probes, the wells were washed with 1×PBS and were further passivated with 0.1% BSA in 1×PBS (100 µL/well) for 30 min at room temperature. Then, streptavidin (10 µg/mL, 100 µL/well) was added to the wells and allowed to incubate for 1 h at room temperature, after which the wells were washed with 1×PBS and incubated with biotin-antibody (10 µg/mL, 100 µL/well) or biotin-ligand (10 µg/mL, 100 µL/well) for 1 h at room temperature. After the incubation, the wells were washed with HBSS and ready for experiments.

Microscopy

Cell free system. Background fluorescence intensity of the glass slides was measured before adding the locks. Lock and lock-HRP strands were added at 200 nM in HBSS to measure the hybridization kinetics. Time-lapse data was acquired in Cy3B channel at 5 positions (The ROI was 81.92 µm×81.92 µm at each position) of each surface and averaged for processing.

Imaging with OT-1 T cells. Around 100,000 purified OT-1 T cells were added to a typical 96 well plate surface and allowed to attach for around 20 min. The cell density was adjusted for the sample surface area. After cells started to produce tension, lock or lock-HRP at 100 nM were added in HBSS for mechanically selective hybridization. After 10 min of incubation, cells were gently rinsed with HBSS and imaged in RICM and Cy3B channels (647 channel is included for cell-free system). For staining experiments, cells were fixed in 4% formaldehyde for 10 min, followed by permeabilization with 0.1% Triton-X for 5 min and blocking for non-specific binding with 1% BSA in 1×PBS. Streptavidin-Alexa488 (SA488) or streptavidin-Alexa647 (SA647) were added to cells at 100 nM overnight at 4 °C to detect biotinylation. After washing with 1×PBS three times, cells were imaged to detect the 488 or 647 signal.

Data analysis. Fluorescence images of the 488, Cy3B and 647 signals were processed by subtracting the camera background, and the fluorescence background of the closed DNA probes using the mean of the local background and 2*SD. The mean intensity was used for quantitative analysis in the cell-free system. The raw integrated intensity was used for the quantitative analysis of the TCR tension, and streptavidin labeling efficiency.

Proximity biotinylation

A stock solution of biotin-phenol was prepared in DMSO at 500 mM and sonicated for 1 min before use. After mechanically selective hybridization was confirmed, biotin-phenol was added to the imaging chamber at 250 µM with or without 1 mM of H_2O_2. After 1 min

of incubation, sodium ascorbate was added at 10 mM (in HBSS) to quench the free radicals. Cells were immediately gently rinsed with HBSS three times to remove excess labeling reagents and quenching buffer. For microscopy experiments, cells were fixed in 4% formaldehyde for 10 min, followed by permeabilization with 0.1% Triton-X for 5 min and then blocking for non-specific binding with 1% BSA in 1×PBS. SA488 or SA647 was added to cells at 100 nM for 30 min at room temperature or overnight at 4 ºC to detect biotinylation. After washing with 1×PBS three times, cells were imaged in the 488 or 647 channels.

For flow cytometry experiments, sodium ascorbate was added immediately after the proximity tagging process, and the cells were gently rinsed three times with HBSS. Ice-cold FACS buffer was gently added, and the cells were quickly scraped into pre-chilled 1.5 mL tubes and kept on ice for 5 min to block the non-specific binding. The SA647 was then added at 50 nM and allowed to incubate for 15 min on ice to detect cell biotinylation. Finally, the cells were spun down and washed twice in ice-cold FACS buffer prior to flow cytometry detection. Flow cytometry experiments were carried out and data was acquired from three biological replicates. Using FlowJo, live OT-1 T cell singlets were isolated by gating based on forward and side scatter area first and then based on forward and side scatter height (**Figure S9**). The geometric mean fluorescence intensity of each sample was calculated and the percent of cells with positive fluorescence was calculated by creating a vertical gate on the fluorescence histogram of the negative control so that ~99.5% of the cells would have fluorescent values less than the value of the gate.

For proteomic and western blot experiments, 75mm×25mm glass coverslips were assembled in the chamber shown below and functionalized with DNA tension probes presenting either antiCD3ε or pMHC N4. Around 10×10^6 naïve OT-1 cells were seeded on to a coverslip and allowed to spread for 10 min. Then, 200 nM of lock-HRP or the unconjugated lock and HRP control were added to cells and allowed to hybridize for 5 min. Microscopy validation was performed to ensure that the cells were exerting tension. The coverslip was then immediately washed with HBSS three times and subsequently added in HBSS containing 250 µM biotin-phenol and 100 µM H_2O_2. After 1 min of incubation, the radicals were immediately quenched with 1 mM sodium ascorbate and 1 mM Trolox and the coverslip was washed three times gently in HBSS. The cells were lysed in ice cold RIPA buffer (with 1×proteinase inhibitor cocktail and 10 mM PMSF) and collected with cell scraper. The lysate was then spun down at 14000×g for 20 min, and the supernatant was collected. Protein quantification was performed with Pierce BCA Protein kit. The lysates were then enriched using streptavidin magnetic beads following the manufacturer's instructions. The beads were snap frozen and stored at -80 °C until further use.

ProPlate® Spring Clip- Tray with 1 chamber assembled:

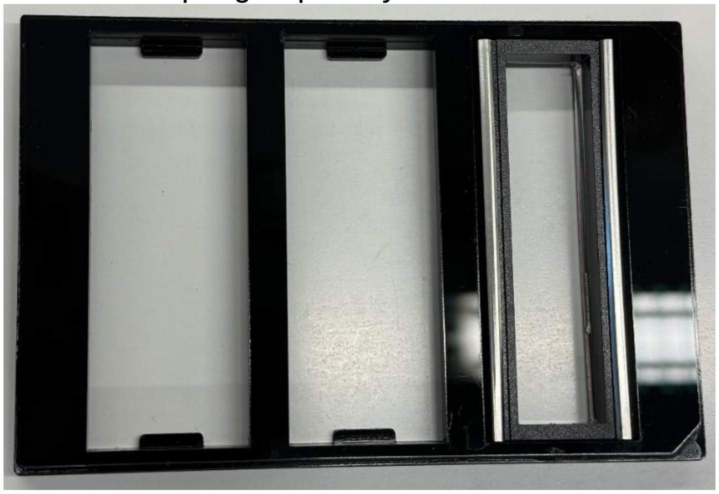

Proteomic Analysis

Western Blot. Beads were mixed with 4×Laemmli buffer (containing β-mercaptoethanol) and denatured at 95 °C for 10 min in thermal cycler. The samples were then subjected to SDS-PAGE electrophoresis and transferred onto a low fluorescence polyvinylidene fluoride (LF PVDF) membrane. The blot was then blocked for 1 h with 5% BSA in TBST for non-specific binding. The blot was subsequently incubated with HRP-conjugated antiTCR, clone H28 at 1:200 dilution in 5% BSA solution for 1 h to stain for TCRα. The blot was rinsed 5 times with 1×TBST after incubation followed by imaging on iBright imaging system. Three biological replicates were performed.

Proteomics sample preparation. Beads were resuspended in denaturing buffer containing 8 M urea, 50 mM Tris-HCl (pH 8.0), and 0.1% DDM, followed by incubation at room temperature for 30 minutes. The urea concentration was subsequently diluted to 1 M using 50 mM Tris-HCl (pH 8.0), after which proteins were reduced and alkylated using 10 mM TCEP and 40 mM chloroacetamide (CAA), respectively. Proteins were digested overnight at 30 °C with 500 ng of Trypsin/Lys-C Mix. Following digestion, samples were acidified with trifluoroacetic acid, and approximately one-fifth of each digest was loaded onto preconditioned EvoTips and processed according to the manufacturer's instructions.

LC-MS/MS. Peptide samples were analyzed on an EvoSep One system coupled to a timsTOF Pro2 mass spectrometer. Peptides were separated on a 15 cm Aurora Elite CSI column (AUR4-150150C18-CSI) using the predefined 40 SPD Whisper Zoom gradient. The mass spectrometer was operated in positive ion mode, utilizing data-independent acquisition with diaPASEF mode[1]. DIA acquisition parameters, including ion mobility and m/z isolation windows, were optimized using the py_diAID tool[2]. The acquisition scheme consisted of 4 ion mobility windows combined with 12 mass isolation windows. Spectra were recorded across an m/z range of 100 to 1700. Fragmentation energies were adjusted based on ion mobility: 20 eV at 0.85 $1/K_0$ and 59 eV at 1.30 $1/K_0$.

Proteomics data analysis. Protein identification and quantification were performed using Spectronaut[3] (version 20) in library-free mode using the Mus musculus SwissProt database (UP000000589; downloaded on 7.1.2022; 17,090 entries) supplemented with the sequence for HRP (UniProt accession number P00433) using default settings. Modifications were defined as follows: Carbamidomethylation (C) as a fixed modification, and Acetyl (Protein N-term) and Oxidation (M) as variable modifications.

All further data processing was performed in R (version 4.4.0 and newer). Only proteins with at least two quantified values in at least one sample group were kept. Intensities were normalized by median centering and log2-scaling. Missing values were imputed by Bayesian PCA imputation[4] and downshift sampling, for proteins missing at random or not at random, respectively[5]. All statistical comparisons between the two groups were performed based on two-tailed Student's t-tests. A p-value below 0.05 was considered statistically significant. Gene enrichment analysis was performed with GOProfiler using their R package, and a custom background was determined based on all identified proteins in the corresponding comparison[6]. Membrane-associated proteins were classified based on subcellular localization annotations from UniProt, specifically selecting entries annotated with the terms "Cell membrane", "Plasma membrane", "cell surface", "Cell membrane", or "Extracellular".

Data availability

The mass spectrometry proteomics data files have been deposited to the ProteomeXchange Consortium via the PRIDE partner repository[7] under project accession: PXD066067.
Reviewer Access under token: 90MwvkGFsSnS.

Alternatively, the reviewer can access the dataset by logging in to the PRIDE website using the following account details:
Username: reviewer_pxd066067@ebi.ac.uk
Password: uMKG0n4btGPC

Figure S1. Preparation of the DNA tension probes substrates. (A) The preparation of gold particle DNA tension probes substrate. (B) The preparation of maleimide and azide modified glass substrates for DNA tension probe immobilization.

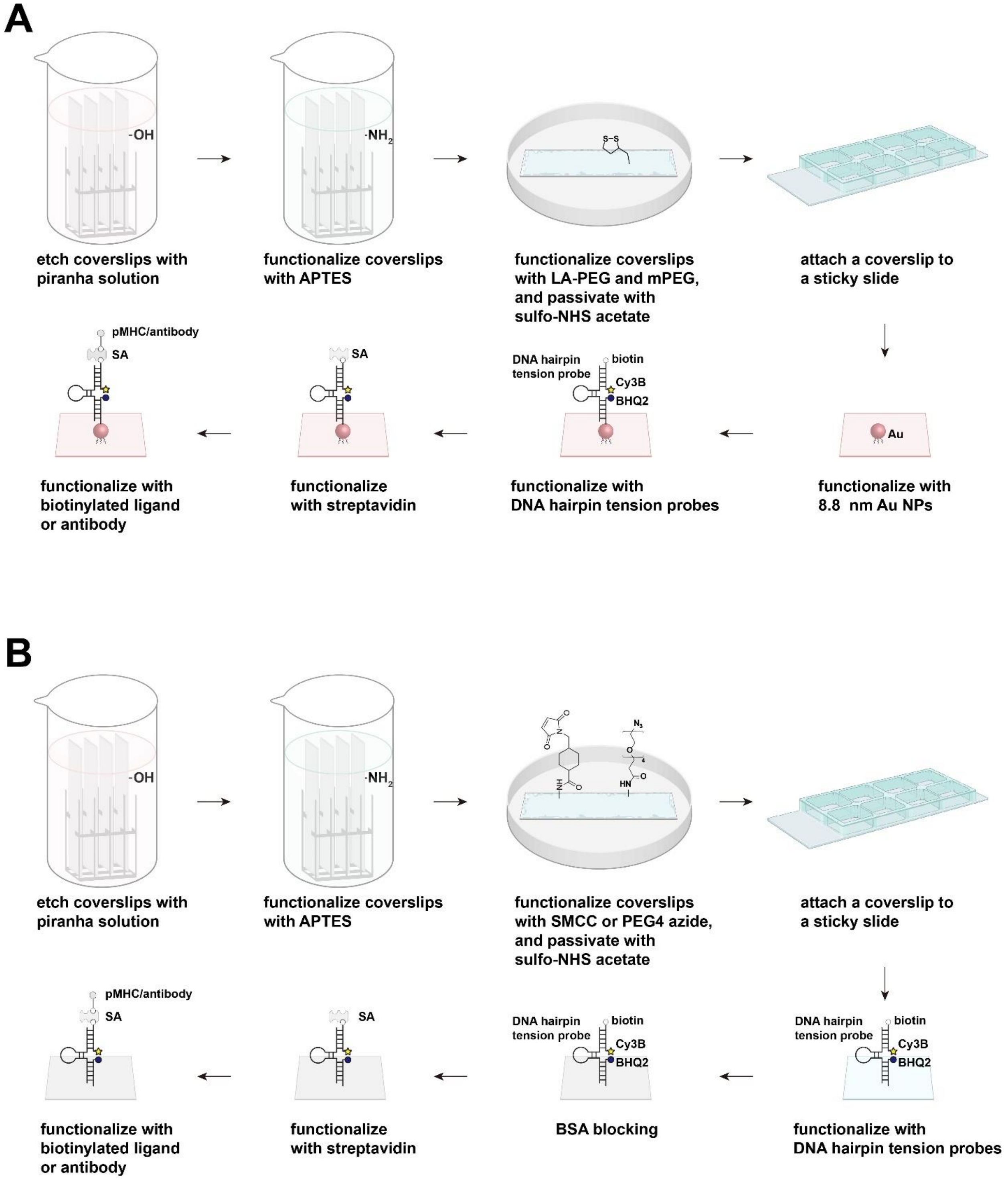

Figure S2. Effect of the conjugation of lock and HRP on in-situ hybridization. (A) Schematic of the binding between the lock and the 4.7 pN DNA tension probes. The goal of this experiment was to determine if HRP conjugation to a locking strand interferes with hybridization kinetics. To address this goal, we used a 17mer lock that invades the hairpin and drives it open. Note that all mechano-ID experiments were conducted with the 15mer lock which fails to significantly open the probe in the absence of TCR forces. Thus, the 17mer was chosen to confirm that the hybridization between the lock and the DNA stem-loop is not affected by conjugation to HRP. (B) Schematic of the hybridization to hairpin probes in the cell-free system. (C) Normalized fluorescence intensity (mean±SD) of the Cy3B signal increase over time with the addition of either 200 nM 17mer, the 17mer lock-HRP, or the non-crosslinked lock and HRP together to the 4.7 pN hairpin probe surface. Fluorescence intensity from multiple positions of the substrates was measured over time, averaged, and normalized to the initial t=0 min intensity.

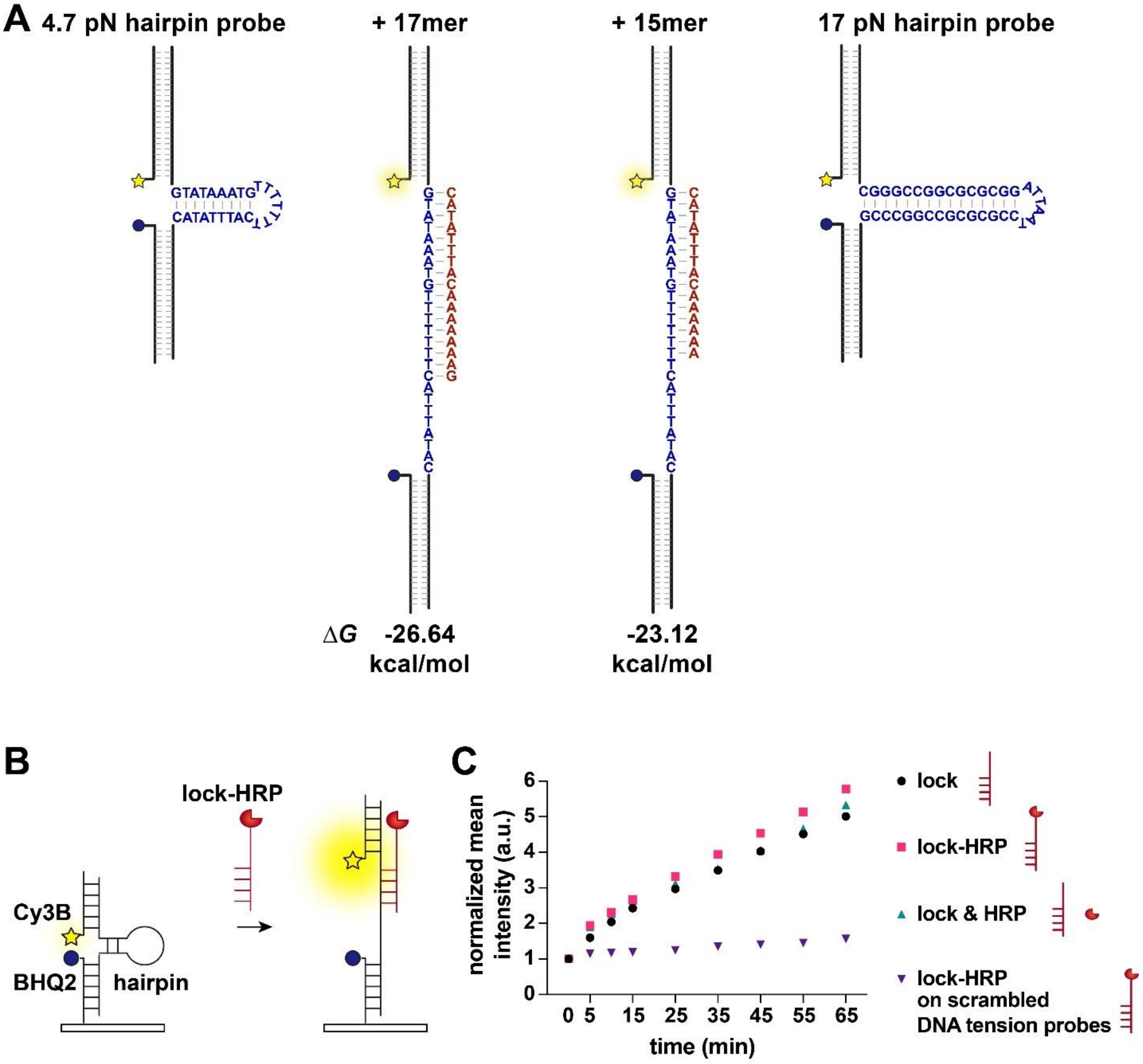

Figure S3. Preparation of oligonucleotides. (A) The general reaction to conjugate amine-modified oligonucleotides with Atto647N NHS. (B) The reaction to conjugate thiol lock to HRP. (C) The reaction scheme to conjugate the lock-HRP with Alexa647 NHS. (D) HPLC trace of A21B Cy3B and lock-647N. The products are marked with arrows. (E) UV-Vis spectra of lock-HRP-Alexa647. (F) Gel electrophoresis image shows the successful conjugation of lock-HRP.

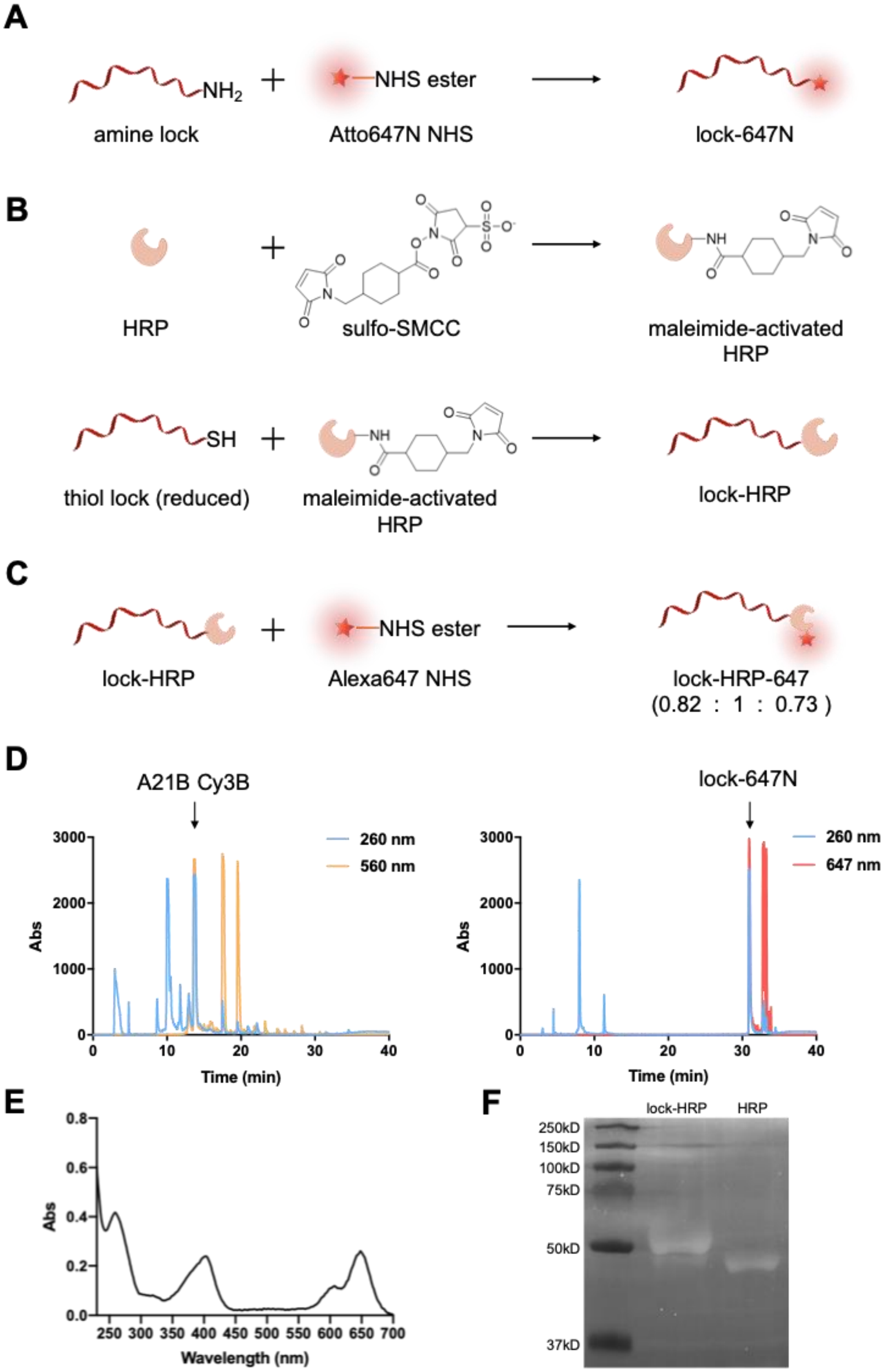

Figure S4. Controls for the proximity tagging of T cells with mechanically active TCRs. (A) Mechanically selective proximity biotinylation with controls of lock&HRP, and lock-HRP on surface with scrambled probe. OT-1 T cells were plated on a DNA tension probe substrate presenting antiCD3ε and allowed to spread for 20 min. After adding the lock-HRP at 100 nM for 10 min, the cells were gently rinsed and supplemented with biotin-phenol and H_2O_2 for proximity biotinylation for 1 min. Then the cells were gently rinsed again and fixed in 4% formaldehyde for 30 min and blocked with 0.1% BSA and stained with SA488. The cells were then imaged in Cy3B channel (indicates fixed tension signal before biotinylation) and Alexa488 channel (indicates biotinylation). Cells on a scrambled 4.7 pN DNA tension probe and cells incubated with non-crosslinked lock and HRP at the same concentration were used as controls for mechanical selectivity of the biotinylation. Scale bar = 5 µm. Only the 4.7 pN DNA tension probe with the complementary lock-HRP showed strong biotinylation. Due to the time lag in between experiment steps, the 4.7 pN tension signal does not always colocalize with the SA488 signal as T cells are highly dynamic. However, this could be minimized by working seamlessly during the experiment to minimize the lag time between adding lock and running the proximity biotinylation reaction.

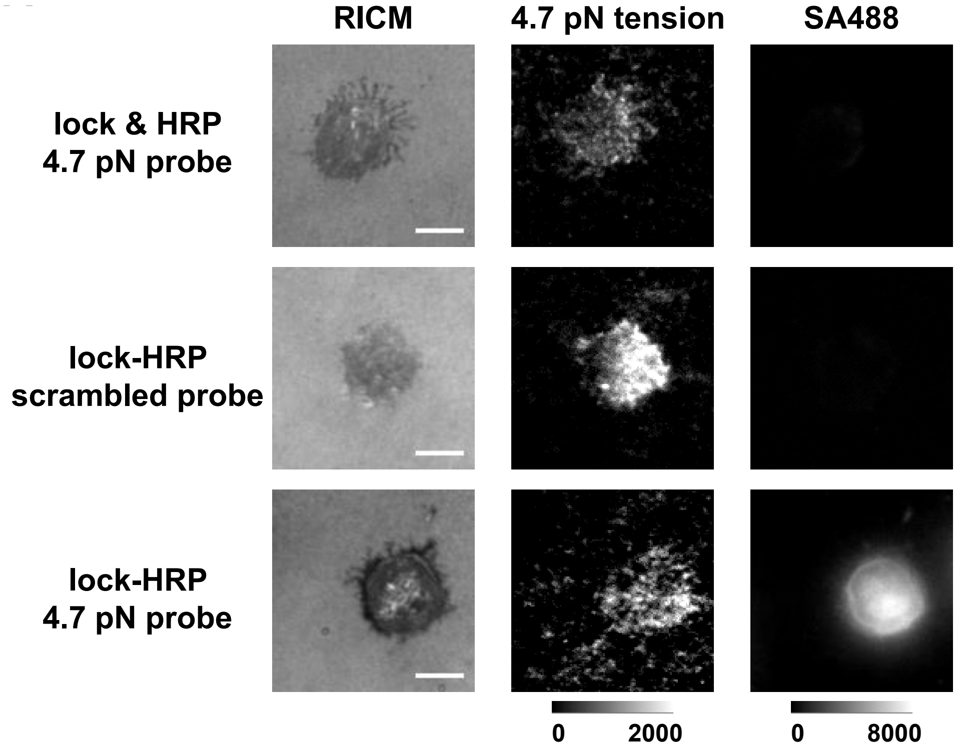

Figure S5. Greater $F_{1/2}$ values lead to reduced proximity tagging. (A) Representative images of RICM, tension signal, and mechano-ID tagging using 4.7 pN and 17 pN DNA hairpins. Surfaces presented pMHC N4 ligands. OT-1 T cells were plated on a DNA tension probe substrate and allowed to spread for 20 min. After adding the corresponding lock-HRP at 100 nM for 10 min, the cells were gently rinsed and supplemented with biotin-phenol and H_2O_2 for proximity biotinylation for 1 min. Then the cells were gently rinsed again and fixed in 4% formaldehyde for 10 min followed by permeabilized with 0.1% Triton-X for 5 min, blocked for non-specific binding with 1% BSA in 1X PBS and stained with 100 nM SA647. The cells were then imaged in Cy3B channel (indicates fixed tension signal before biotinylation) and Alexa647 channel (indicates biotinylation). Scale bar = 5 µm. 17 pN DNA tension probe showed weaker SA647 signal compared to 4.7 pN DNA tension probe surfaces. (B) Quantification of tension signal and proximity biotinylation between 4.7 pN and 17 pN DNA tension probes. Each data point shows the intensity per cell, experiments were performed in triplicate and the plot shows mean +/-SD. The groups were compared using a one-way ANOVA.

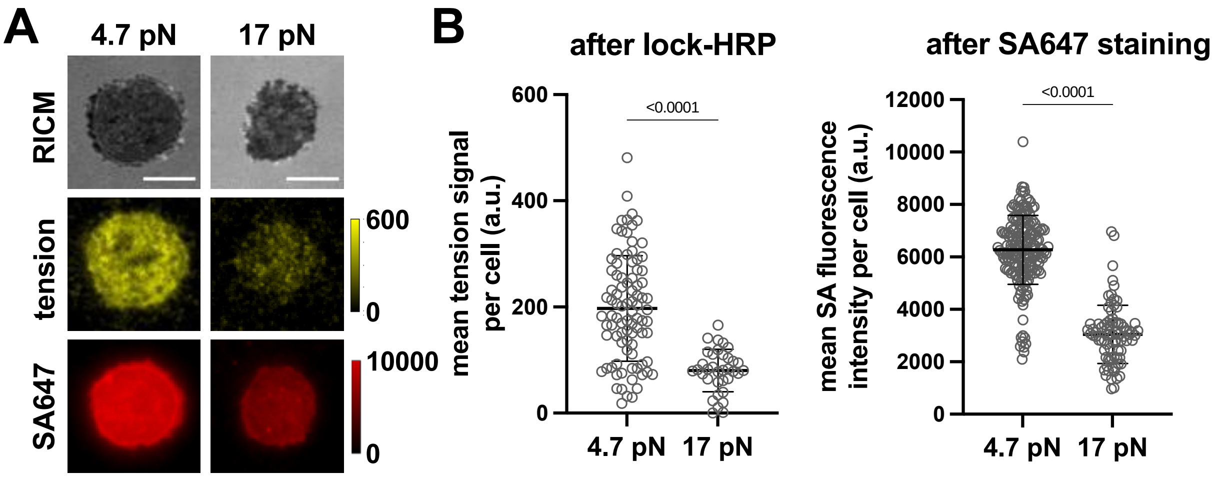

Figure S6. Heterogeneity of tension among T cells. (A) Representative images showing the spreading of the OT-1 naïve $CD8^{+}$ T cells on DNA tension probe substrate presenting pMHC N4, and producing tension, which was locked by 250 nM lock-HRP for 5 min. Cells with more frequent mechanical sampling of the pMHC N4 showed higher tension signal and vice versa. Scale bar = 10 µm. (B) Histogram of the distribution of OT-1 contact area on pMHC N4 DNA tension probes. (C) Histogram of the distribution of the OT-1 TCR tension integrated intensity after 5 min incubation with lock-HRP.

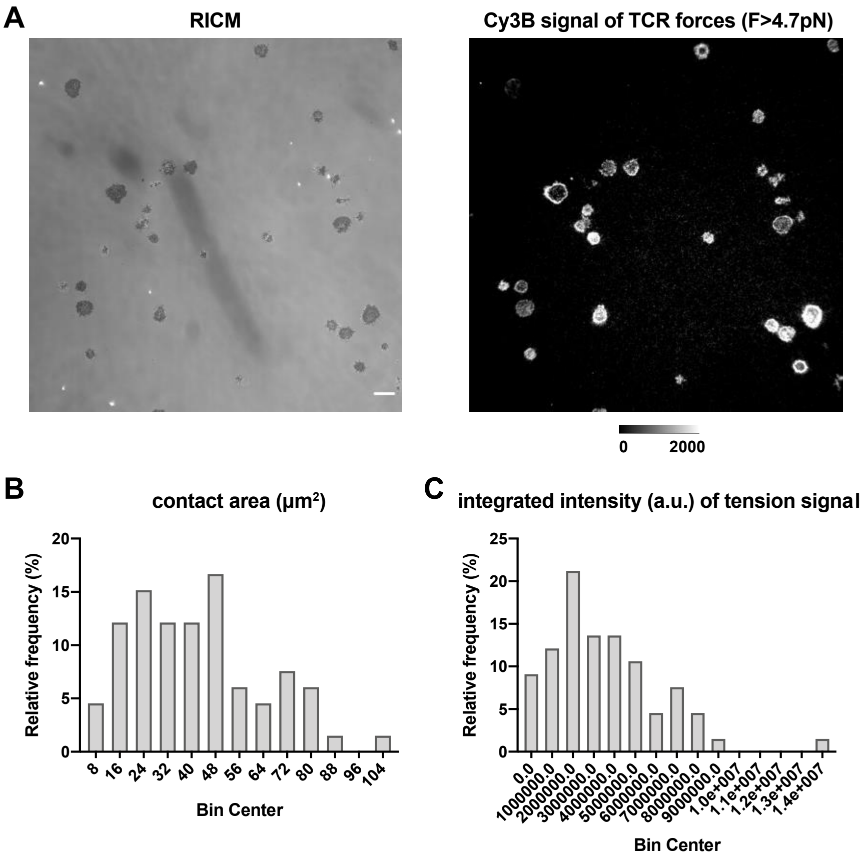

Figure S7. Comparison of different approaches for cell collection. (A) RICM imaging was used to evaluate cell release following DNase I treatment. We found that cells exposed to the proximity tagging procedure failed to detach from the surface. OT-1 T cells were allowed to engage the DNA tension probe substrate (with or without proximity biotinylation) and then treated with DNase I at 0.5 mg/mL for 5 min at room temperature (imaging buffer contains 1.2 mM Ca^{2+} and 0.8 mM Mg^{2+}). We did not investigate this observation further, but we speculate that the oligonucleotides undergo modification due to the phenoxyl radical and these modified nucleotides are more resistant to DNase I. (B) Bright field images of cells engaged with tension probe surfaces, and then subjected to mechano-ID with H_2O_2 and without H_2O_2 treatment. These images show that H_2O_2 does not alter cell attachment density. (C) RICM of OT-1 T cells showing scraping is an efficient and effective cell collection method. Scale bar = 10 µm.

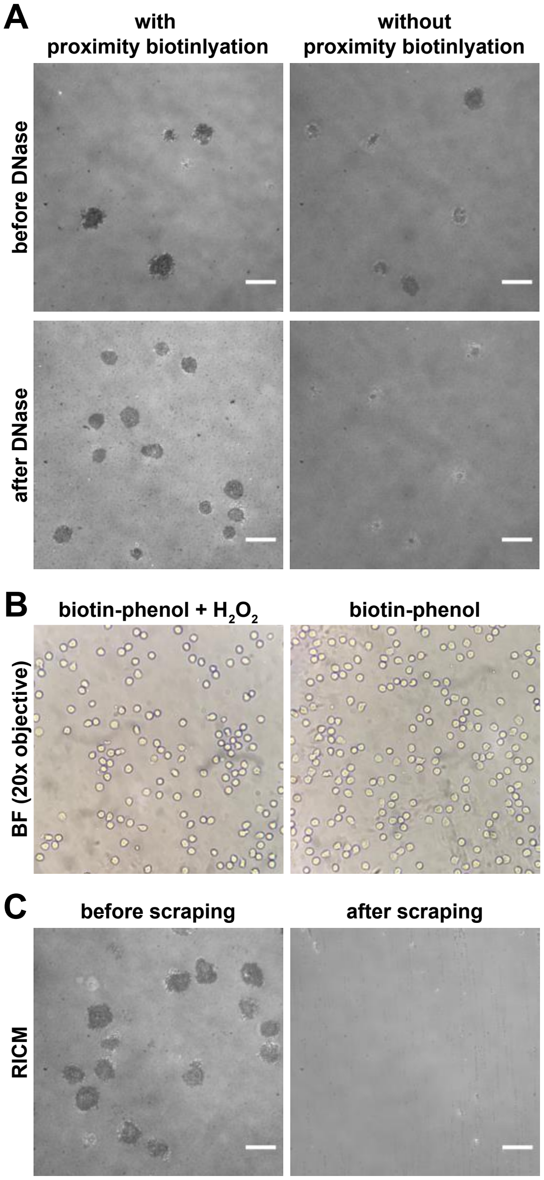

Figure S8. Representative RICM and tension images for pMHC Q4 and pMHC N4 CD8 blocked samples. (A) Raw microscopy images of tension signals (Cy3B) from pMHC Q4 and pMHC N4 CD8 blocked probes before and after locking using same contrast as shown in **Figure 4**. Scale = 10 μm. (B) Raw microscopy images of tension signals (Cy3B) from pMHC Q4 and pMHC N4 CD8 blocked probes before and after locking and after changing the contrast.

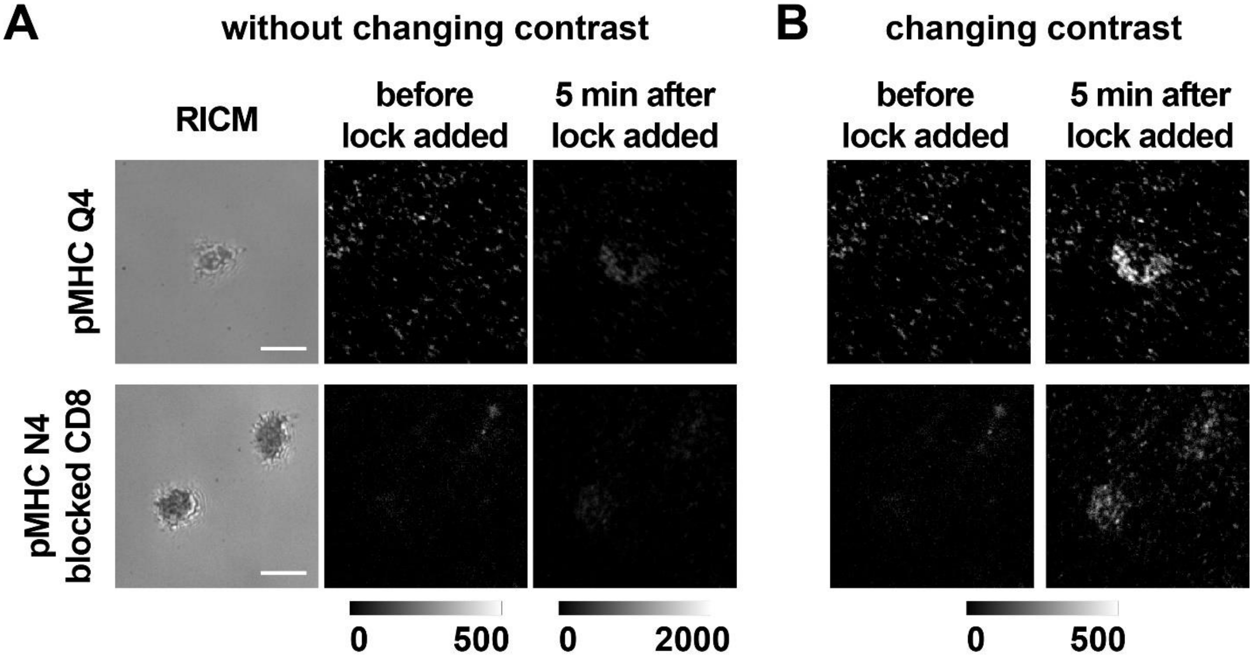

Figure S9. Representative gating of live singlet T cells with forward scatter and side scatter plots. Flow analysis of mechano-ID tagged cells was performed as described in the methods section. We used the forward scatter/side scatter profile to gate out cell debris and cell aggregations as shown below. Only live singlet cells were analyzed in the flow cytometry data.

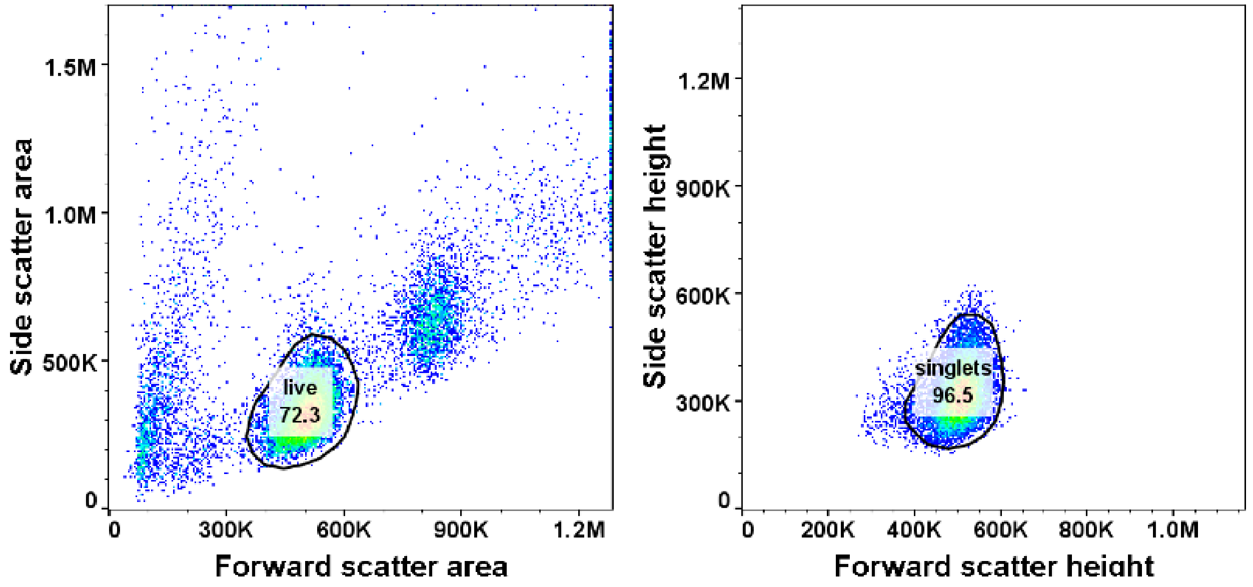

Figure S10. Representative images of gels and western blots. (A) Protein gel of samples before streptavidin enrichment. (B) Protein gel of samples after streptavidin enrichment. Protein gels are stained with SYPRO Tangerine fluorescent protein stain to visualize total proteins from cell lysates before transfer onto western blot membrane. (C) Western blot of samples before streptavidin enrichment. (D) Western blot of samples after streptavidin enrichment. Blots were immunostained with HRP-conjugated anti-TCRα (H28) followed by chemiluminescence labeling to detect biotinylated proteins. Experiments were performed in triplicate.

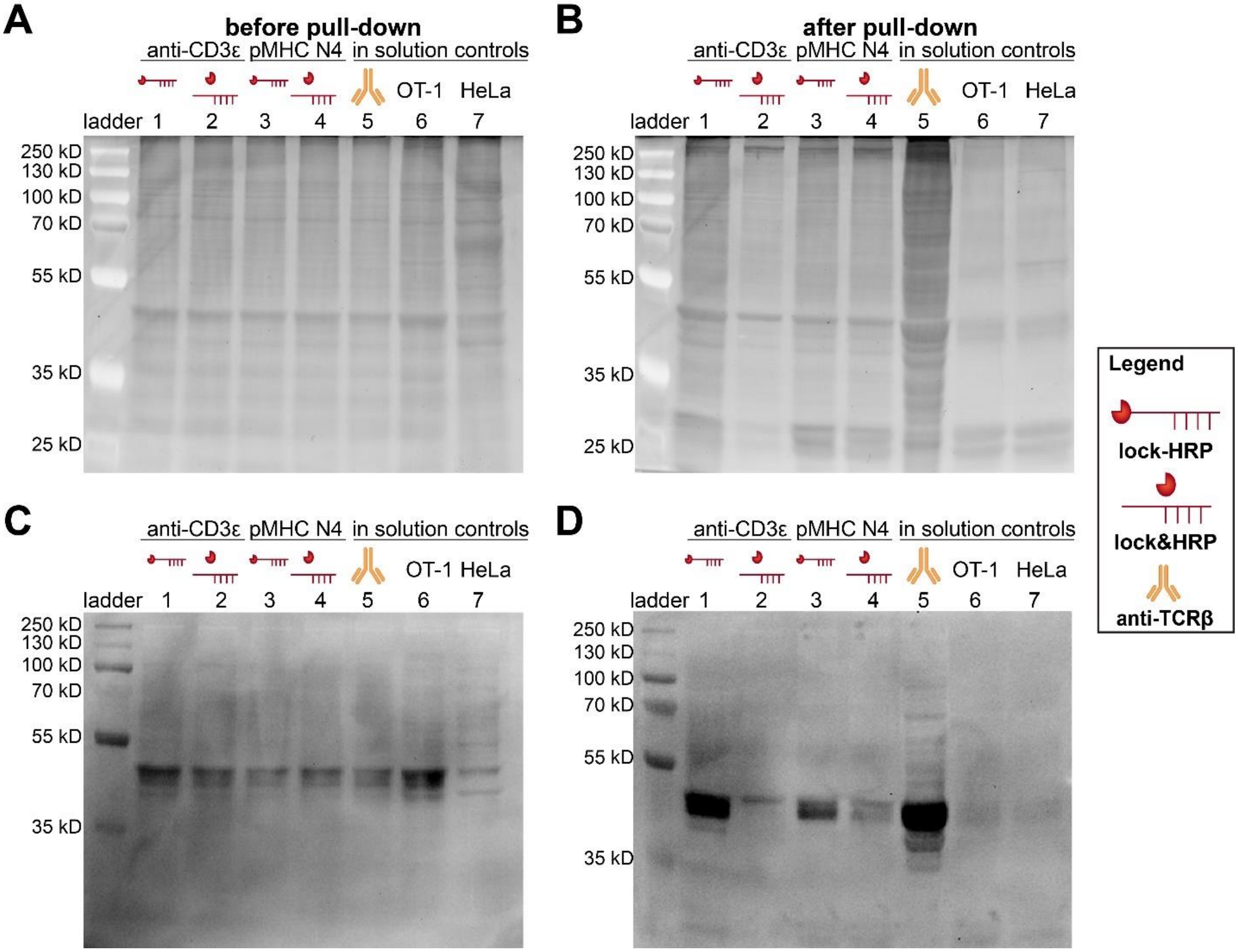

Figure S11. Proteomics profiling of enriched proteins after mechanically selective proximity labeling. (A) Schematic diagram for proteomic experiments, including microscopy validation. (B) Representative image of RICM and tension signals (Cy3B) using 4.7 pN hairpins. Microscopy imaging was performed as a validation step to ensure naïve OT-1 T cells are exhibiting tension signals prior to performing mechano-ID followed by proteomic analysis. Scale bar = 5 µm. (C) Density plots of antiCD3ε and pMHC N4 experiments indicating high enrichment of cell membrane proteins over other proteins. (D) Illustration of known nearby interactome of TCR-CD3 complex upon TCR binding. (E) Box plots of known extracellular and intracellular proteins that are associated with TCR engagement. Box plots show data sets for both antiCD3ε and pMHC N4 probes. Extracellular proteins showed increase in fold change while intracellular proteins showed minimal difference or decrease in fold change between lock-HRP and lock&HRP (negative) for both antiCD3ε and pMHC N4 probes. Fold change and p values are shown in **Table S4**.

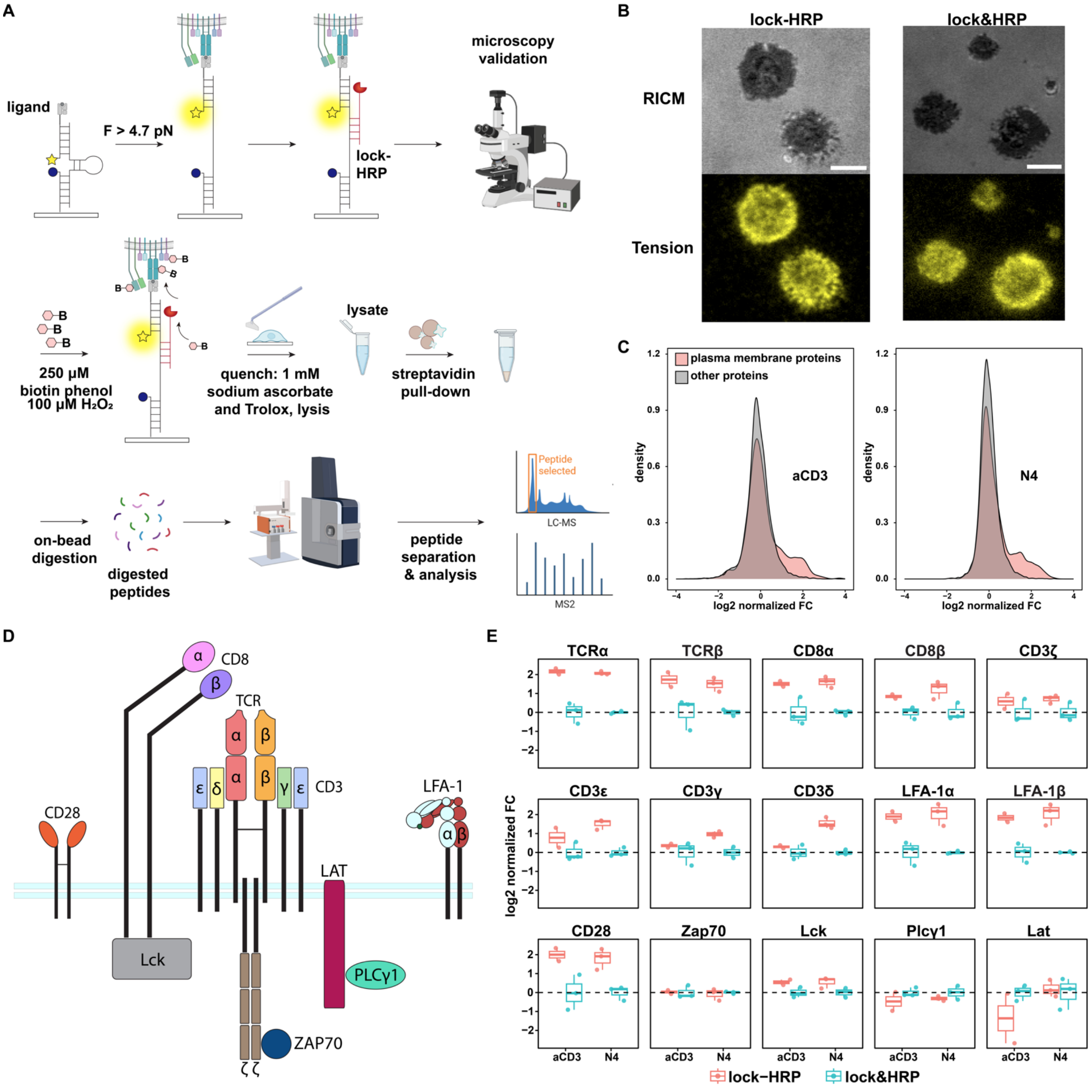
A
ligand
F > 4.7 pN
lock-HRP
microscopy validation
250 μM biotin phenol 100 μM H2O2
quench: 1 mM sodium ascorbate and Trolox, lysis
lysate
streptavidin pull-down
on-bead digestion
digested peptides
peptide separation & analysis
Peptide selected
LC-MS
MS2
B
lock-HRP
lock&HRP
RICM
Tension
C
plasma membrane proteins
other proteins
density
aCD3
N4
log2 normalized FC
D
CD8
TCR
CD3
CD28
LFA-1
LAT
Lck
PLCγ1
ZAP70
E
TCRα
TCRβ
CD8α
CD8β
CD3ζ
CD3ε
CD3γ
CD3δ
LFA-1α
LFA-1β
CD28
Zap70
Lck
Plcγ1
Lat
log2 normalized FC
aCD3
N4
lock−HRP
lock&HRP

Figure S12. Comparison of labeled membrane proteins by antiCD3ε and pMHC N4 mechano-ID probes. (A) 2D volcano plot with y-axis portraying log2 fold change for antiCD3ε and x-axis portraying log2 fold change for pMHC N4 experiments. The top right quadrant represents the proteins that are enriched in both sets. Top 20 overlapped differentially enriched proteins are highlighted in red and labeled. Total number of similar & unique differentially enriched proteins between antiCD3ε and pMHC N4 probes indicated on Venn diagram. (B) Box plots of top 20 differentially enriched proteins from (A). Box plots show data sets for both antiCD3ε and pMHC N4 probes. Fold change and p values are shown in **Tables S5, S6** and **S7**. Experiments were performed in triplicate except for antiCD3ε (lock-HRP).

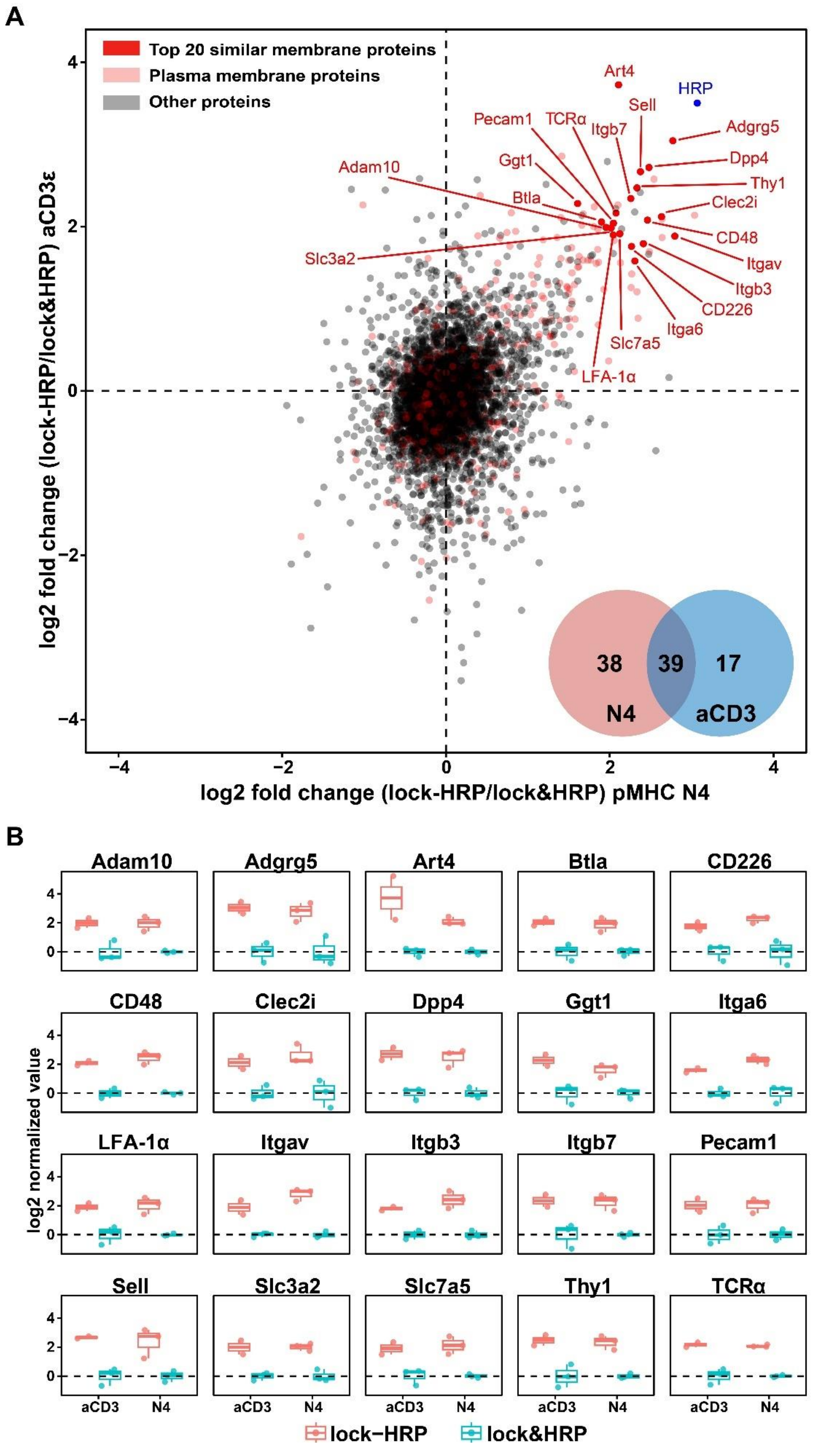
A
Top 20 similar membrane proteins
Plasma membrane proteins
Other proteins
Art4
HRP
Sell
Adgrg5
Pecam1
TCRα
Itgb7
Dpp4
Ggt1
Thy1
Adam10
Btla
Clec2i
CD48
Slc3a2
Itgav
Itgb3
CD226
Itga6
Slc7a5
LFA-1α
38
39
17
N4
aCD3
log2 fold change (lock-HRP/lock&HRP) aCD3ε
log2 fold change (lock-HRP/lock&HRP) pMHC N4
B
Adam10
Adgrg5
Art4
Btla
CD226
CD48
Clec2i
Dpp4
Ggt1
Itga6
LFA-1α
Itgav
Itgb3
Itgb7
Pecam1
Sell
Slc3a2
Slc7a5
Thy1
TCRα
log2 normalized value
aCD3
N4
lock−HRP
lock&HRP

Table S4. List of TCR-CD3 complex members and canonical proteins known to be associated with TCR engagement enriched in both antiCD3ε and pMHC N4 ligands.

Protein Accessions	Gene Name	antiCD3ε		pMHC N4	
		p value	FC	p value	FC
P01849	Trac/TCRα	0.01448174	2.165227223	5.72E-06	2.077545199
P01851	P01851/TCRβ	0.08369517	1.7320536	0.003199234	1.485898648
P01851	CD8α	0.072571749	1.50593168	0.001037882	1.610056707
P10300	CD8β	0.043739493	0.838528048	0.031579892	1.224642287
P24161	CD247/CD3ζ	0.364662846	0.585576478	0.08432255	0.707863221
P22646	CD3ε	0.239297216	0.78016463	0.001911272	1.511150558
P11942	CD3γ	0.491674272	0.360573284	0.007839175	0.961867071
P04235	CD3δ	0.383358835	0.298113356	0.001132646	1.545200277
P24063	Itgal/LFA-1α	0.033722406	1.898616758	0.003885523	2.043106407
P11835	Itgb2/LFA-1β	0.021721114	1.831779056	0.003351607	2.054412994
P31041	CD28	0.072707635	2.000945526	0.009636651	1.804413117
P43404	Zap70	0.925381458	0.026403839	0.68259588	-0.07997422
P06240	Lck	0.094556796	0.540539488	0.068234063	0.552422658
Q62077	Plcγ1	0.332199349	-0.470841535	0.23144648	-0.315201389
O54957	Lat	0.276500225	-1.362505342	0.739202901	0.193501168

Table S5. List of similar membrane proteins enriched in both antiCD3ε and pMHC N4 ligands and their associated GO terms. p values and FC shown are obtained with pMHC N4 ligand. Cutoff p value<0.05 and FC>0.75.

Gene Name	Biological Process	p value	FC
Adam10	T cell activation; molecular scissors cleaving surface proteins	0.002909835	1.955813558
Adgrg5	role is under investigation	0.015306629	2.771319966
Art4	Immunoregulation; ADP-ribosyltransferase	3.67E-04	2.107286463
Atp1a1	Immunoregulation; regulate T cell exhaustion	0.001698973	1.622751418
Atp1a2	role is less understood	0.00234946	1.626599592
Atp1b1	Immunoregulation; regulate T cell exhaustion	0.004714145	1.535348393
Btla	Immunoregulation; inhibitory coreceptor of BCR	0.003836476	1.899670999
Ccr7	T cell activation; co-stimulatory receptor	0.011843227	1.510598573
CD226	T cell activation; co-activating receptor	0.011244087	2.265991408
CD44	Adhesion and migration	0.034744057	1.280830102
CD48	Immunoregulation and T cell activation	6.77E-04	2.461373866
CD5	Immunoregulation; inhibitory receptor	0.003407287	1.482739543
CD84	Immunoregulation	0.006824599	1.676954215
CD8β	T cell activation; co-receptor of TCR	0.031579892	1.224642287
Clec2i	T cell activation	0.017285223	2.63220514
Dpp4	T cell activation; co-stimulatory molecule	0.003973239	2.479906235
Ggt1	Immunoregulation; oxidative stress & apoptosis	0.008078514	1.606988295
Ifnar1	Immunoregulation	0.02794092	1.121880353
Igf2r	Immunoregulation; insulin growth factor signaling	7.50E-04	0.752721451
Itga6	Adhesion and migration	0.004280679	2.308006212
Itgal	Adhesion and migration	0.003885523	2.043106407
Itgav	Adhesion and migration	5.55E-04	2.795226912
Itgb1	Adhesion and migration	2.33E-04	2.332931633
Itgb2	Adhesion and migration	0.003351607	2.054412994
Itgb3	Adhesion and migration	0.003150934	2.411075816
Itgb7	Adhesion and migration	0.002494945	2.257409813
Ly6c2	Adhesion and migration	0.001448561	1.857335951
Ly6d	role is under investigation	0.011421787	1.766554468
Ly9	Immunoregulation; inhibitory molecule	0.00469638	1.732750074
Pecam1	Adhesion and migration	0.004925967	2.044966445
Ptprc	Immunoregulation	0.001183711	1.853792916
Sell	Adhesion and migration	0.019835388	2.375509002
Sema4a	T cell activation; co-stimulatory molecule	0.007137325	1.835784352
Sema4d	T cell activation; co-stimulatory molecule	0.019036162	1.01443686
Slc3a2	T cell activation; amino acid transporters	0.001856138	2.017169701
Slc7a5	T cell activation; amino acid transporters	0.004146707	2.123840463
Thy1	Immunoregulation; co-stimulatory molecule	0.001413648	2.3331342

TCRα(Trac)	T cell activation; stimulatory receptor	5.72E-06	2.077545199
Vsir	Immunoregulation; negative regulator	0.003637479	1.675380337

Table S6. List of unique membrane proteins enriched in antiCD3ε ligands and their associated GO terms. Cutoff p value<0.05 and/or FC>0.75.

Gene Name	Biological Process	p value	FC
Adi1	No known role in T cell immune response	0.039532836	-1.362432675
CD47	Immunoregulation; negative regulator	0.01348808	1.127816405
Dag1	T cell survival	0.019081844	1.212139736
Flot2	Immunoregulation; negative regulator	0.023471089	0.980756638
Jaml	T cell activation; costimulatory molecule	0.029731156	1.589654015
Kcnn4	Immunoregulation; calcium signaling	0.015423524	0.828326557
Lpar5	Immunoregulation; inhibitory molecule	0.002666119	1.612311688
Lrrc8c	Immunoregulation; inhibitor of T cell activity	0.002787596	1.011909738
Mib1	T cell development; regulator of Notch signaling	0.037542802	1.888044737
Minpp1	No direct role in T cell immune response	0.040920292	-1.062592368
Ptprs	Immunoregulation; inhibitory molecule	0.010912134	1.624154021
Sh3bgrl	Role is less understood; adapter protein	0.01798793	-2.073727257
Slc27a1	Role is under investigation; fatty acids transporter	0.037379257	-0.750922119
Slc39a10	T cell survival; zinc transporter	0.035699606	1.762083354
Slc44a1	Role is under investigation; choline transporter	0.018401758	1.42394255
Slc7a1	T cell activation; amino acid transporters	0.010873467	-1.744364617
Thbs1	T cell activation; inhibitor molecule	0.021674422	2.16801657

Table S7. List of unique membrane proteins enriched in pMHC N4 ligands and their associated GO terms. Cutoff p value<0.05 and/or FC>0.75.

Gene Name	Biological Process	p value	FC
Adam17	Immunoregulation	0.011734024	1.393089633
Adgre5	Adhesion and migration	0.017982133	1.509047584
Ano6	Immunoregulation	0.008469688	1.172937989
Apobr	Role is under investigation	0.044294104	1.498842611
Art2b	Immunoregulation; ADP-ribosyltransferase	0.001266954	3.037316281
CD2	Adhesion; costimulatory molecule	0.010112319	1.539250399
CD27	T cell activation; costimulatory molecule	0.038201239	1.539437512
CD28	T cell activation; costimulatory molecule	0.009636651	1.804413117
CD3ε	T cell activation; signal transducer	0.001911272	1.511150558
CD6	Immunoregulation; costimulatory molecule	0.004847426	1.17164327
CD8α	T cell activation; co-receptor	0.001037882	1.610056707
Clca3a1	role is less understood	0.008895796	1.658550295
Clec2d	Immunoregulation; inhibitory receptor	0.005106802	2.099832713
Cpm	T cell activation	0.022010151	1.404123913
Emb	Cell adhesion and migration; nutrient transport chaperone	0.001461559	2.484209727
Enpp1	Immunoregulation	0.035292394	1.987272216
G3bp1	Immunoregulation	0.01466496	1.082159165
Ifnar2	Immunoregulation & T cell development	0.00272443	2.346967884
Ifngr1	T cell activation	0.001672232	1.576769553
Igf1r	Immunoregulation; insulin growth factor signaling	0.002804728	1.35827243
Il1rap	Immunoregulation	0.012793991	2.261605323
Il2rb	T cell activation	0.044772633	1.277397611
Il6st	Immunoregulation	0.025073002	1.389168481
Insr	Immunoregulation; stimulatory receptor	5.02E-04	1.478699468
Itga5	Cell adhesion and migration	0.003709554	1.569702132
L1cam	Immunoregulation; immunosuppression	0.025174337	2.639709251
Lair1	Immunoregulation; inhibitory receptor	0.002041674	1.864329412
Nptn	T cell activation	0.013410844	2.640305875
P10404	Immunoregulation; immunosuppression	0.01898629	1.13039649
Plaur	Immunoregulation	0.030555664	1.927905737
Plxna1	T cell activation	0.022493888	1.49623246
Ptpra	Cell adhesion; positive promoter	3.67E-04	1.127523774
Serinc3	role is under investigation	0.004628144	2.540255336
Slamf1	Immunoregulation	0.049379118	1.403823945
Slamf6	Immunoregulation	0.003201642	1.81188841
Slc44a2	role not fully understood; fatty acid transporter	0.035540149	0.911900015
Tfrc	T cell activation; involved in iron uptake	0.012849667	1.861637488
Usp8	Immunoregulation	0.001411551	-0.789286565

References

1. Meier, F. *et al.* diaPASEF: parallel accumulation-serial fragmentation combined with data-independent acquisition. *Nat Methods* 17, 1229-1236 (2020).

2. Skowronek, P. *et al.* Rapid and In-Depth Coverage of the (Phospho-)Proteome With Deep Libraries and Optimal Window Design for dia-PASEF. *Mol Cell Proteomics* 21, 100279 (2022).

3. Bruderer, R. *et al.* Optimization of Experimental Parameters in Data-Independent Mass Spectrometry Significantly Increases Depth and Reproducibility of Results. *Mol Cell Proteomics* 16, 2296-2309 (2017).

4. Jin, L. *et al.* A comparative study of evaluating missing value imputation methods in label-free proteomics. *Sci Rep* 11, 1760 (2021).

5. Lazar, C., Gatto, L., Ferro, M., Bruley, C. & Burger, T. Accounting for the Multiple Natures of Missing Values in Label-Free Quantitative Proteomics Data Sets to Compare Imputation Strategies. *J Proteome Res* 15, 1116-1125 (2016).

6. Kolberg, L. *et al.* g:Profiler-interoperable web service for functional enrichment analysis and gene identifier mapping (2023 update). *Nucleic Acids Res* 51, W207-W212 (2023).

7. Vizcaino, J.A. *et al.* ProteomeXchange provides globally coordinated proteomics data submission and dissemination. *Nat Biotechnol* 32, 223-226 (2014).
